# Rapid telomere motions in live human cells analyzed by highly time-resolved microscopy

**DOI:** 10.1186/1756-8935-1-4

**Published:** 2008-10-27

**Authors:** Xueying Wang, Zvi Kam, Peter M Carlton, Lifeng Xu, John W Sedat, Elizabeth H Blackburn

**Affiliations:** 1 Department of Biochemistry and Biophysics, University of California at San Francisco, California, USA; 2 Molecular Cell Biology, Weizmann Institute of Science, Rehovot, Israel; 3 Genome Institute of Singapore, Singapore

## Abstract

**Background:**

Telomeres cap chromosome ends and protect the genome. We studied individual telomeres in live human cancer cells. In capturing telomere motions using quantitative imaging to acquire complete high-resolution three-dimensional datasets every second for 200 seconds, telomere dynamics were systematically analyzed.

**Results:**

The motility of individual telomeres within the same cancer cell nucleus was widely heterogeneous. One class of internal heterochromatic regions of chromosomes analyzed moved more uniformly and showed less motion and heterogeneity than telomeres. The single telomere analyses in cancer cells revealed that shorter telomeres showed more motion, and the more rapid telomere motions were energy dependent. Experimentally increasing bulk telomere length dampened telomere motion. In contrast, telomere uncapping, but not a DNA damaging agent, methyl methanesulfonate, significantly increased telomere motion.

**Conclusion:**

New methods for seconds-scale, four-dimensional, live cell microscopic imaging and data analysis, allowing systematic tracking of individual telomeres in live cells, have defined a previously undescribed form of telomere behavior in human cells, in which the degree of telomere motion was dependent upon telomere length and functionality.

## Background

Telomeres, essential for protecting chromosome ends [1-3], consist of tandem telomeric DNA repeats bound by multiple proteins that collectively 'cap' the telomere (reviewed in [4]). A minimum length of telomeric repeats is necessary to support this protective function. Telomeric repeats are replenished by the cellular ribonucleoprotein enzyme, telomerase [2,5]. Most human cancer cells have high telomerase activity. In contrast, normal human cells have naturally limited levels of telomerase that can lead to telomere shortening over time. Many aspects of telomeres remain incompletely understood, especially their dynamic properties and behavior over time. At one extreme, over the decades of human lifespan, telomeres generally shorten, with rates and extents that have been associated with disease progression and risk [6-9]. Here we report new findings on telomere behavior at the other end of the timescale spectrum: the dynamics of individual human telomeres in living cells analyzed at 1-second time resolution.

The dynamics of the nuclear contents are complex and determined by multiple contributing factors. First, the diffusion of non-interacting particles experimentally inserted into the nucleoplasm is much slower compared with their diffusion in water [10]. The apparent diffusion coefficient of these free inserted particles has strong size-dependence, beyond that predicted by Stokes' Law [10,11]. This deviation from the values expected from Stokes' Law has been attributed to the characteristic free space between structural elements. In the specific case of a polymer molecule, the diffusion within entangled concentrated polymers is described as snake-like reptation motion confined by a tube formed by the other polymer molecules [12]. Reptation is orders of magnitude slower than diffusion in dilute solutions. Secondly, the dynamics of chromatin within the dense nucleus are even further slowed down, probably due to chromatin architecture and its specific interactions. Chromosomal dynamics include oscillatory-like fast motions as well as apparently diffusive motions. Labeled chromatin sites move with seconds timescale oscillations [13], as well as showing constrained diffusion [11,13,14]. This motion is highly variable for different regions within the same nucleus or even the same chromosome ([15,16], and references cited therein). This variability has been related to interactions with nuclear structures (nuclear envelope [17], nuclear pores, nucleoli [18], promyelocytic leukemia (PML) and Cajal bodies [19-21], nuclear matrix, DNA-associated proteins, telomeres [14,22]) and to the conformation of the chromatin itself (centromeres [23], heterochromatin, DNA unwinding at highly expressed genes, histone acetylation [10], DNA methylation and cell cycle-related chromosome condensation [24]). In this study, we focused on short timescale, apparently diffusive, motion of the telomeres. We did not attempt to explain theoretically the diffusion coefficient values measured. Rather, we concentrated on differences found between telomeres and their relation to other measured properties (notably position and size) and to controlled perturbations.

A recently developed microscopy platform was employed. This platform (and variants of it) is applicable for a variety of cell-biological observations. Here we applied it to analyze rapid telomere dynamics, in order to compare the motions of telomeres of different known lengths and functionality. As the present study focused on short timescales, where the motion is described closely by diffusion laws, the motions are expected to reflect telomere interactions relatively directly. We report that in living cells, telomeres exhibit novel, heterogeneous rapid motions that directly reflect impaired telomere functionality.

## Results

### Generation of cell lines with fluorescently labeled telomeres

**Figure 1:**
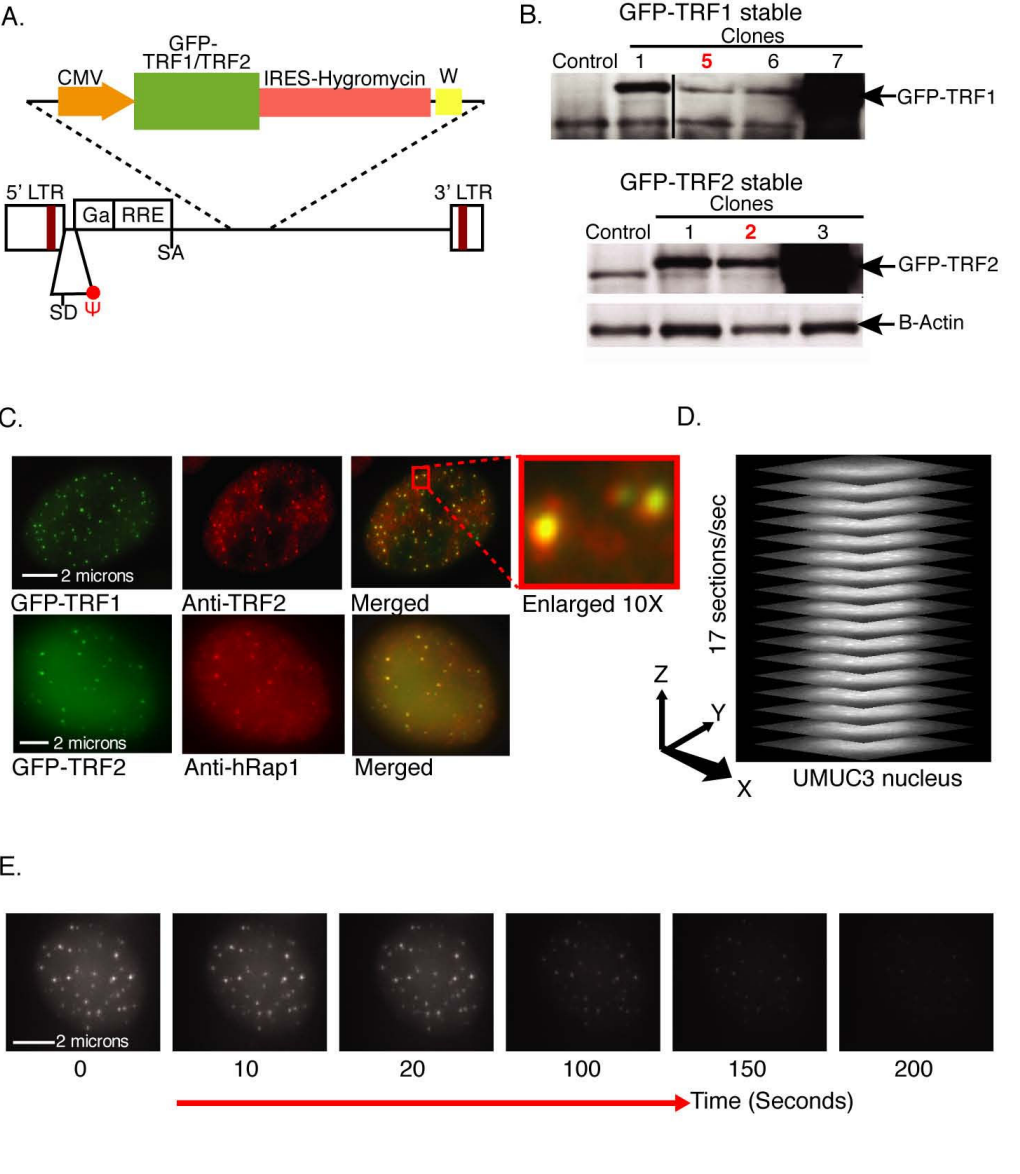
**Visualization of telomeres in live cultured cancer cells**. (A) A schematic of lentivector expressing green fluorescent protein (GFP)-tagged TRF1 or TRF2. The vector map is referenced in the Methods section. (B) Western blotting analysis of UMUC3 cells stably expressing GFP-TRF1 or GFP-TRF2 using antibodies against TRF1 or TRF2. Clonal lines with the lowest average expression (indicated by red numbers) were chosen for comparison with the endogenous level of TRF1 or TRF2 for telomere dynamic analysis. (C) Indirect immunofluorescence of clonal UMUC3 cell lines expressing either GFP-TRF1 (upper row) or GFP-TRF2 (lower row). Complete colocalization was observed from GFP-TRF1-expressed telomeres stained with anti-TRF2 antibody in fixed cells in the upper row. Lower row: immunofluorescence images of GFP-TRF2-expressed telomeres stained with anti-hRap1 antibody also showed telomere colocalization. (D) A schematic of the data collection regime for a representative UMUC3 nucleus. One complete three-dimensional (3-D) stack of 8 μm deep (an entire UMUC3 nucleus) with 0.5 μm focal spacing (17 sections) was acquired every second. (E) Representative examples of projection images from one complete 3-D stack of GFP-TRF1-expressed UMUC3 nucleus that was acquired every second for 200 consecutive seconds (*T*, time in seconds). The photobleaching curve is shown in D in Additional file 1.

Telomeres were visualized using two of the major telomeric binding proteins, TRF1 or TRF2, fluorescently labeled as green fluorescent protein (GFP) fusion proteins (Figure 1A) and expressed at low levels. The GFP tag moiety *per se *expressed in cells does not confer motility, as GFP-histone H2B shows very little movement in nuclei [25]. For these analyses, we focused on a bladder carcinoma cell line, UMUC3, because it has relatively short telomere lengths (ranging from around two to five kilobase pairs), typical of human tumor cells. Stably transfected clonal lines were generated. For minimal perturbation of telomere properties, the clonal lines with the lowest expression levels of the fusion GFP-telomeric protein were chosen for telomere dynamics analyses (Figure 1B, A in Additional file 1). Individual telomeric signals within a nucleus had the expected large variation in their intensities and spot sizes. These parameters were reflective of the relative magnitudes and known intra-cell variability [26] of telomere lengths in cancer cells, as seen in both the UMUC3 cell line (Figure 1C, Additional File 1) and the other cell lines analyzed. Several criteria, described below and throughout the paper and discussed below, established that the fluorescent spots analyzed were bona fide telomeres. First, the cell lines analyzed in the present study were telomerase-positive cancer cell lines and not cell lines with alternative lengthening of telomeres (ALT), in which ALT-associated PML bodies occur [27,28], and in which some PML body dynamics have been reported [14,29]. The total numbers of fluorescence points per nucleus were counted and fitted well with the number of chromosome ends, and were independent of the expression level of the tagged telomeric proteins. Colocalization experiments using pair-wise combinations of three different telomeric proteins, TRF1, TRF2 and hRAP1, also showed the expected patterns of colocalization and intensity correlations expected for individual telomeres (Figure 1C).

### Acquisition and quantitative analyses of images with high temporal resolution

High-resolution, three-dimensional (3-D, *xyz*) spatial image datasets were acquired within rapid timeframes (100 ms for a stack of 17 consecutive focal planes with 0.5 μm focal spacing, encompassing the entire 6–8 μm high nucleus, every second for 200 seconds) using an optical microscope platform (OMX) (Figures 1D and 1E, Additional file 2). By analyzing local motion over such short times, we avoided the complexities of reaching the constrained motion thresholds found in yeast, *Drosophila *and mammalian nuclei [11,13,18], and could apply diffusion equations to quantify the telomere motion tracks as a function of time.

Two separate software programs (Track4D and TrackIt4D) were custom written to analyze the image datasets for large numbers of cells. Typically, per UMUC3 nucleus, 80 or more spots were resolved, of which 40 to 60 unambiguous telomeres could be tracked for extended periods within each total 200-second data collection timeframe (see C-E in Additional file 1). The total numbers of identifiable telomeres were consistent with those expected if the great majority of fluorescent spot signals are from single telomeres. Hence in these cells, unlike telomeres in yeast [30,31], it is unlikely that in general, multiple telomeres cluster into single spots. For the experiments reported here, the complete four-dimensional (4-D) datasets (3-D and time) for every nucleus were displayed and analyzed in six different ways and are shown as appropriate throughout this paper in one or more of the following ways: (1) as movies (Additional files 3, 4, 5); (2) as kymographs – plots showing the movement of each individual telomere as its position in the *xy*-plane as a function of time *T *(see A and B in Additional file 6, Additional files 7 and 8); (3) as a trajectory of each telomere, showing its position in 3-D space as a function of time for 200 consecutive seconds (see E in Additional file 1, C in Additional file 6, Additional files 7 and 8); (4) as the end-to-end (E2E) distance in 3-D space as a function of time (each line in the plot tracks the distance of an individual telomere at time *T *seconds away from its original starting point at time 0) (see D in Additional file 6, Additional files 7 and 8); (5) as the cumulative path distance traveled by a telomere between time 0 and time 200 seconds (see E in Additional file 6, Additional files 7 and 8); (6) as the average E2E distance for each telomere for the time interval traveled (see F in Additional file 6, Additional files 7, 8, 9). E2E measurements were used to calculate *D*, the effective diffusion coefficient, according to Einstein's diffusion equation, E2E = (6*DT*)^1/2 ^(*T *is the time in seconds). Hence telomere motion was quantified for every telomere and the population of telomeres, both in a single cell and in multiple cells.

### Heterogeneity of telomere motion

**Figure 2:**
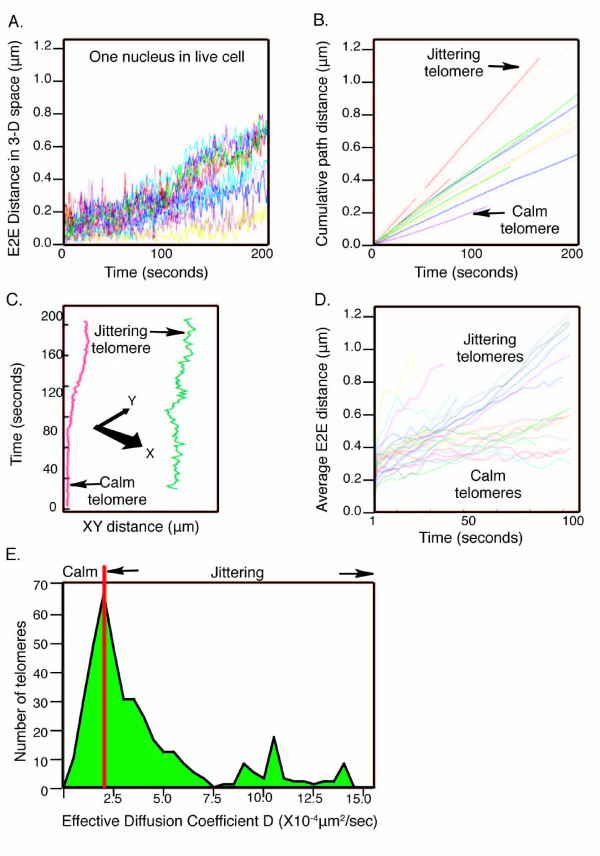
**Heterogeneous motions of telomeres**. Telomeres tracked using green fluorescent protein (GFP)-TRF1 are shown as representatives. GFP-TRF2 telomeric tracks are shown in A in Additional file 10. (A) Tracking of telomere motion by the software program Track4D, shown as end-to-end (E2E) distance in three-dimensional (3-D) space. An individual telomere has moved in space away from its 0 time-point position. Hence this displays motion of each telomere as a function of time *T *(seconds), and its distance (μm) from its starting point in the first timeframe. The effective diffusion coefficient, *D*, was calculated from these E2E measurements. (B) TrackIt4D tracking of telomere motion as demonstrated by plotting of the cumulative path distance traveled between time 0 and time *T *(seconds). (C) Tracking from kymographs (3-D tracks projected on to a *xy *plane) of a live UMUC3 cell showing one representative of a calm telomere and a jittering telomere. (D) Telomere motion statistics calculated from average E2E distance over sub-intervals. All distances traveled by the same telomere over time intervals of the same length were averaged together and plotted. Telomeres tracked for 200 seconds were plotted for intervals up to 100 seconds. (E) Telomere motion quantified as a histogram of *D *values, *N *= around 400 telomeres.

In unperturbed UMUC3 cells within a single live cell nucleus, individual telomeres moved independently and showed a large variability of motion (Figure 2A–D, A in Additional file 10, Additional file 3). As shown in the plots of cumulative path (Figure 2B, E in Additional file 6, Additional files 7 and 8, A in Additional file 11, B in Additional file 12, E in Additional file 13, D in Additional file 14), over the 200-second measurement period, for all telomeres tracked, the accumulated path maintained the same line slope: the fast-moving telomeres kept moving fast, and the slower telomeres kept moving slowly. Hence, there was no indication of any change in the trend of motion that might have been attributed to photobleaching effects.

The mean and median of these UMUC3 cell telomeric *D *values fell in the general range for diffusion coefficients previously reported for chromatin segments in internal chromosome regions [11,13,32] (see A and B in Additional file 6, Additional files 7 and 8). Notably, the histogram based on around 400 telomeres exhibited a left-skewed distribution of *D *values with its mode at around 2.3 ± 0.6 × 10^-4 ^μm^2^/second (Figure 2E). We estimated the error bars in such histograms using a non-parametric statistics bootstrap procedure (see B in Additional file 10). When the histograms were plotted with a log scale for the *D *values, the distribution still showed asymmetrical skewing (shown in C in Additional file 10 for the data in the histogram in Figure 2E). This indicates that the skewing seen in Figure 2E does not describe an underlying additive or multiplicative random process. Based on the modal value, we arbitrarily defined the sub-set of telomeres having an average *D *value of less than 2.3 × 10^-4 ^μm^2^/second as 'calm' telomeres (moving less than 0.37 μm during 100 seconds tracking). Within the same nucleus, other telomeres, defined as 'jittering' telomeres, moved back and forth rapidly and irregularly (some having *D *> 10^-3 ^μm^2^/second, corresponding to their moving on average more than 0.77 μm during 100 seconds) (Figure 2C and 2E, Additional file 3).

**Figure 3:**
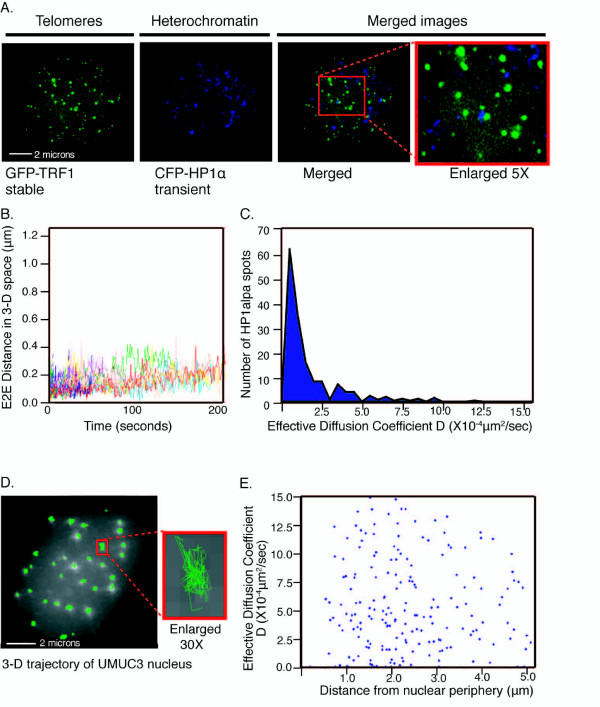
**Characterization of heterochromatin and telomere motion**. (A) Colocalization of telomeres using green fluorescent protein (GFP)-TRF1 with heterochromatin stained by CFP-HP1α shows that most telomeres do not colocalize with the heterochromatin foci in UMUC3 cells. (B) Track4D analysis of heterochromatin motion in three-dimensional (3-D) space shows that the UMUC3 cell heterochromatin moves with much less motion than UMUC3 cell telomeres. (C) Histogram of *D *values for heterochromatin motion, *N *= around 400 heterochromatin spots. Heterochromatin hardly moves, and *D *values for heterochromatin fall in the range reported (mode around 1 × 10^-4 ^μm^2^/second). (D) 3-D trajectory image of tracked telomeres in a UMUC3 nucleus. The enlarged view shows the motion path of a representative jittering telomere and its non-directional movement and telomeric motion conforms to a 'constrained non-directional walk' description. (E) A scatter plot of diffusion coefficients of telomeres against distance from the periphery shows that there is no significant association of telomere movement with the nuclear periphery, and no correlation between distance to the periphery and motion characteristics.

We asked how the variability of telomeric motion, and in particular that of the more rapidly moving telomeres, compared with the mobility of another class of heterochromatic regions. To mark such internal regions along chromosome arms in the same cell background, we analyzed live UMUC3 cells transiently transfected with HP1α, a non-histone protein that binds to certain heterochromatin regions [33]. This produced a punctate fluorescence pattern (Figure 3A), consistent with the reported localization of HP1 proteins to heterochromatic regions in human HeLa cells [13,18,34]. Immunofluorescence staining of the CFP-HP1α protein revealed that the majority of the spots did not colocalize with telomeres (Figure 3A). Hence we could distinguish telomeric signals from this class of chromosome-internal signals. Analysis of the dynamics of these HP1 heterochromatin loci was carried out as for telomeres. Consistent with reports on heterochromatin regions in yeast, *Drosophila *and mammalian models (*D *values of 0.05–1.3 × 10^-4 ^(m^2^/second)) reviewed in [15] and [16]; see A and B in Additional file 6, Additional files 7 and 8), the HP1α heterochromatin foci were less motile than the telomeres in the same UMUC3 nucleus (Figure 3B), with significantly smaller and more uniform effective diffusion coefficients (*D *values: mode around 1 × 10^-4 ^μm^2^/second) (Figure 3C, A in Additional file 11). Thus, notably, the modal *D *values for telomeres were twice as high as for the HP1α-containing heterochromatic class of internal positions on chromosomes. However, most striking was the much greater range of telomeric motions compared with the HP1α-positive spots; some individual telomeres had diffusion coefficients over 10 times greater still (see B in Additional file 11). The possibility that the fastest telomeric fluorescence spots were free telomeric protein particles was ruled out because free particles with comparable sizes have *D *values in nuclei thousands of times greater than those measured here for the fastest-moving telomeres [35] (see A in Additional file 15). These and other findings described below excluded the possibility that the telomeric fluorescence signals came from free protein aggregates. Therefore, based on these findings, and those described below, the widely heterogeneous motion is not a property shared with the specific HP1α-containing class of heterochromatic regions at internal positions on chromosome arms. However, variability in motion of internal chromosomal loci has been reported in a study examining the relative motions of two fluorescently marked loci within a nucleus [18]. These studies (which were done with a time-point resolution of 1 minute instead of the 1-second resolution in the present work) showed that the motions of the chromosome internal loci examined were more constrained when they were in proximity to the nucleolus or nuclear periphery [18].

The telomere motion can be described as 'a constrained non-directional walk'. Unlike non-constrained free diffusion, which predicts a linear relationship between the square of the distance traveled by a particle, the mean square distance (MSD), and the time duration *T *[32,36], the MSD curve in the internal regions of a chromosome generally reaches a plateau of *T *> 200 seconds in yeast and mammalian cells [11,18]. This is interpreted as 'constrained diffusion'. However, our telomeric analyses, performed for only 200 seconds, were done before the E2E distances (around 0.37 μm) reached any evident chromosome territory confinement limit, estimated to be 0.6 μm in mammalian and other eukaryotic nuclei [11,32]. Therefore, below 200 seconds, the assessment of telomere movement by the effective diffusion coefficient is justified (see A in Additional file 16). The movement of individual telomeres was also clearly not directional (Figure 3D). In yeast, telomeres and centromeres are tethered at or near the nuclear envelope [22] and reviewed in Gasser [37], with obvious implications for telomere motility. As mentioned above, a study on the motion of non-telomeric loci in mammalian cells (examined with a 1-minute time resolution) found that these motions were more constrained when the loci were close to the nuclear periphery [18]. In contrast, no attachment of telomeres to the nuclear periphery has been reported in mammalian cells. Consistent with this, we observed that telomere motion was statistically no lower at the peripheral region of a nucleus than deep in the same nucleus (Figure 3E). This is shown by inspection of the nuclear localization of individual telomere trajectories, approximating the nucleus as an ellipsoid and using the Mahalanobis distance from the center to define proximity to the nuclear periphery (see B in Additional file 16). Hence, jittering and calm telomeres may occur randomly throughout the nucleus.

### Effect of azide treatment on telomere motility

**Figure 4:**
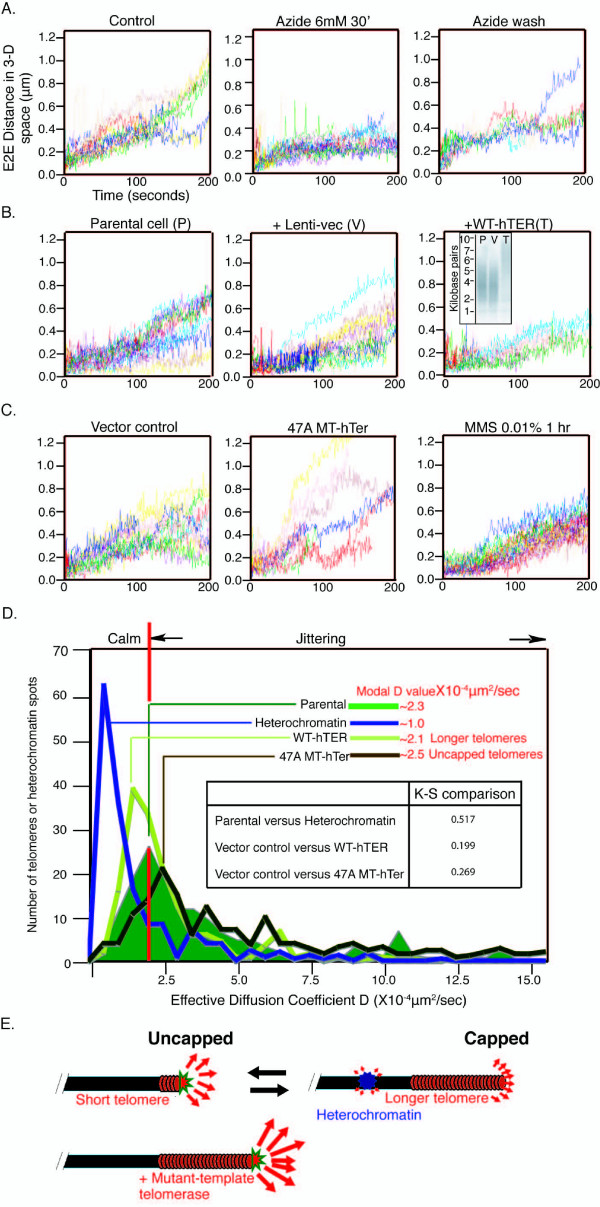
**Telomere movement can be altered experimentally**. (A) Azide treatment of UMUC3 cells shows that jittering telomere movements require energy. Washing away azide partially restores the movement of jittering telomeres. (B) Southern blotting (insert in right panel) shows that UMUC3 telomeres were extended by expressing an extra telomerase RNA (WT-hTER) in cells, which dampens the telomere movement (right panel). The empty vector does not change the telomere motion (middle panel). (C) 47A MT-hTer, which induces DNA damage foci colocalizing with telomeres (see E in Additional file 14), increases telomere motility in UMUC3 cells (middle panel), whereas the general DNA damaging drug methyl methanesulfonate does not (right panel). (D) A summary of histogram analysis of parental telomeres, longer telomeres under WT-hTER treatment, uncapped telomeres under 47A MT-hTer treatment, or heterochromatin (*N *= around 400 telomeres or heterochromatin spots). The Additional file 18 insert shows that the Komolgorov-Smirnov non-parametric comparison between histograms is statistically significant. (E) A model for the novel form of the dynamic telomere behavior reported here: short telomeres and uncapped telomeres have higher motility than longer, functionally capped telomeres or other internal regions of chromosome arms. The motility of each mobile chromosomal entity is indicated by the length/strength of the arrow.

The higher rates of motion (jittering) of individual telomeres required energy. A brief azide treatment (6 mM for 30 minutes) dramatically and reversibly reduced the motion of the faster telomeres, while the position of the modal *D *value stayed the same (Figure 4A, A-C in Additional file 12). Various potential spurious contributions to motility, such as nuclear volume changes, nuclear drift, or rotation, were ruled out as described in B in Additional file 12; see also Additional file 4.

### Greater telomere motility is associated with shorter telomeres

Two types of observations suggested that the more rapid telomere motion was related to telomere shortness. First, in three other human cancer cell lines with different mean telomere lengths, we noted that the longer the average bulk telomeric DNA length, the lower the telomere motions (see A and B in Additional file 13). This relationship between mean telomere spot signal intensity and telomeric DNA length within (see below) and across different cell types provided additional corroborative evidence that the signals were bona fide telomeres. Second, comparing telomere motions within each single UMUC3 nucleus, there was an inverse relationship between telomere fluorescence intensity and motion: short telomeres, identified as those with lower intensity values, contributed the majority of the jittering telomeres (see C and D in Additional file 13). Finally, plotting the entire data for a collection of 20 different parental UMUC3 cells in a clonal sub-line expressing GFP-tagged TRF1 shows an inverse linear relationship between the effective diffusion constant (*D*) of a telomere with signal intensity, across the spectrum of telomeric signal intensities (except at the extreme upper signal intensity boundaries) (see Additional file 17). These data showed that in these UMUC3 cells, a two-fold decrease in telomere length (as shown by the telomere signal intensity) corresponded to a 2.7-fold increase in effective diffusion coefficient.

We tested whether there was a causal relationship between telomere shortness and higher telomere motion. To change the average bulk telomere length with the minimal perturbation possible while keeping the same cell background, we stably expressed telomerase RNA (WT-hTER) above its endogenous level in UMUC3 cells [38]. As shown, this extends telomeres moderately, by three to four kilobase pairs, in these cells (Figure 4B). Telomeres became less motile and more homogeneous in their movement than in the parental cells or cells that had received the control empty vector. The quantitative changes in motility were evident from two different parameters: both the modal *D *value and the number of highly motile telomeres decreased (see E-G in Additional file 13). The great majority of the telomeres (as shown by the position of the modal peak) became shifted to the lower *D *value when the great majority of the telomeres in the UMUC3 cells had been shifted to a longer telomeric DNA tract length (the mean telomere DNA length seen in the Southern blot in Figure 4B). The cell doubling time was the same in all these cells (parental, vector-control, and WT-telomerase RNA overexpressing cells), as reported previously [39]. Together, these results ruled out the possibility that a shift in the cell population cell cycle could account for the difference seen. Hence, shifting the bulk telomere population length upwards dampened telomere motility.

### Experimental uncapping of telomeres increases telomere motility

Even with adequate telomerase, as in the human cancer cells analyzed here, telomeres cycle between capped and uncapped states as a normal part of their maintenance [40]. It was proposed that such temporarily uncapped, often shorter, telomeres are those acted on by telomerase [3,41], thereby replenishing their length and restoring their ability to be capped. We hypothesized that the greater motility of shorter telomeres in the experimental settings described above could reflect such a partially uncapped status. We therefore tested whether uncapping of telomeres by an independent means could cause increased motion. We induced rapid and irreversible telomere uncapping by an independent means: expressing a mutant-template telomerase RNA (MT-hTer). Such MT-hTers cause telomerase to add terminal mutant repeats, which are designed to be unable to bind sequence-specific protective telomeric proteins. MT-hTers were shown to rapidly de-protect (uncap) telomeres and accumulate colocalizing DNA damage foci on telomeres [38,39,42]. Notably, MT-hTers uncap telomeres without detectable shortening of the rest of the telomeric DNA tract or depletion of bulk telomeric proteins TRF1 or TRF2 from the telomeres [38,39,42]. Such expression of an MT-hTer ('MT-hTer 47A') in UMUC3 cells greatly increased the fraction of jittering telomeres, as seen by kymograph analyses, their expanded trajectories and large E2E and *D *values (Figure 4C, A-F in Additional file 14). We eliminated the possibility that this exaggerated telomere movement induced upon telomere uncapping is a result of the general DNA damage response state of these cells. This was done by examining telomere motions in cells treated with a DNA alkylating agent, methyl methanesulfonate (MMS), which produces comparable numbers of DNA damage foci that do not colocalize with telomeres (see E in Additional file 14). Under MMS treatment, telomeres remained as restrained in their movement as the controls (Figure 4C). Measurement of nuclear volumes upon these treatments established that cell/nuclear volume shrinkage or expansion did not occur, excluding this contribution to changes in telomere motions. Hence, uncapping of telomeres by MT-hTer 47A increases telomere motion; in contrast, treatment with MMS did not.

## Discussion

The ability of the OMX to collect 4-D live datasets with high time resolution (every second for a short timeframe of 200 seconds) has allowed examinations of large numbers of data points for statistically robust analyses on telomere dynamic behavior. This enabled evaluation of the apparent telomere diffusion coefficient without the complications of long-term constraint diffusion imposed by the restriction of chromosomes to limited spatial nuclear domains. In this setting, we have discovered sub-sets of telomere dynamics within human cancer cell nuclei. Previous work has shown energy dependence of the motions of PML bodies in live human cancer cells, but such previous analyses have not addressed the rapid high-resolution motions of validated telomeres [14,29].

A novel finding is that chromosome end motion is highly heterogeneous and inversely proportional to telomere length. Furthermore, experimentally uncapping telomeres, but not the general DNA damage caused by MMS treatment, greatly increased telomeric motions (Figure 4D, Additional file 18). It is possible that the telomeric uncapping resulting from incorporation of mutant repeats causes the increased telomeric motion and that DNA damage at other genomic locations in general does not. Alternatively, MMS damage may engage a different DNA response pathway from that elicited by telomere uncapping.

Even in untreated cells there was a wide range of telomere motility, with the shorter telomeres more motile on average than longer ones in the same nucleus. Experimentally increasing the telomerase activity level, with concomitant lengthening of the telomeres, damped down their movement and reduced the number of jittering telomeres in the population. Although it could be conceived that longer telomeres could allow more binding sites for (unspecified) protein attachments, such load addition to just a few kilobase pairs of telomere length would be insignificant considering the size of the chromosome. We therefore propose the model (Figure 4E) that short and, therefore, uncapped telomeres, or telomeres uncapped by mutant repeat addition, with resultant loss of protection, become more motile and move at a greater speed, which enables them potentially to explore and possibly sample a larger volume in the nucleus. The greater motility could occur during or upon uncapping as a result of telomeres detaching from a nuclear sub-structure, or active (and hence potentially energy-dependent) decompaction of a relatively large sub-telomeric (heterochromatic) region, hence altering the elasticity of this region and allowing greater freedom of telomere movement. The data also are consistent with the possibility that telomeres may not specifically attach to an immobile structure in the nuclear matrix, since one specific class of internal arm regions of chromosomes moved slower than telomeres.

Telomeres and their replenishment machinery including telomerase have elicited interest as possible targets for use in anti-cancer therapies [43]. Therefore, understanding the basic behavior of single telomeres in living cells will be an important underpinning of any use of such potential therapeutics. Furthermore, the critically short telomeres in non-cancerous cells without sufficient telomerase eventually become uncapped, triggering cellular senescence and apoptosis, which are thought to contribute to aging and mortality (reviewed in Shay and Wright [44]). Tracking the very rapid, seconds-scale time events at telomeres in cells in real time will thus provide more understanding of the heterogeneity of the dynamics of this complex biological system.

## Conclusion

The examination and systematic analysis of telomere dynamics at 1-second time intervals for 200 consecutive seconds reported here has shown that chromosome ends show distinct movements that are different from chromosome-internal HP1α heterochromatin regions. Specifically, individual telomeres within a single live cell nucleus displayed wide heterogeneity in their degrees of movement over short time intervals, and quantitative analyses show that such telomere motion consists of a 'constrained non-directional walk'. The class of chromosome-internal heterochromatin regions examined in this study showed less, more homogeneous motion. Short telomeres and experimentally 'uncapped' telomeres had higher motility than longer telomeres. Conversely, experimentally extending telomere length lowered telomere motility. Hence, integrating physical/mathematical and biological approaches has shown new properties of telomeres reflective of telomere functionality. The systems developed here to accurately and statistically track telomeres should be generally applicable to any punctate chromatin (or cellular) foci in live cells.

## Methods

### Lentiviral constructs and plasmids

The N-terminus of the TRF1 and TRF2 cDNAs were fused in-frame with enhanced GFP (Clontech, Mountain View, CA, US). The C-terminus of the TRF1 and TRF2 cDNAs were fused with internal ribosome entry site-hygromycin (IRES-hygromycin; Clontech, Mountain View, CA, US). The resultant fusion constructs were sub-cloned into the pHRCMVGFPWSin18 vector under the CMV promoter (courtesy of Dr Didier Trono) [45]. The CFP-HP1α was a kind gift from Dr Tom Misteli [33]. WT-hTER and 47A MT-hTer in lentiviral vectors were generated as in Li et al [38]. Briefly, WT-hTER was polymerase chain reaction-cloned from human genomic DNA and sub-cloned into the *Blg*II/*Sal*I site in pIU1-T7 plasmid (generously provided by Dr Edouard Bertrand). MT-hTer was then generated by site-directed mutagenesis to create an hTer with the RNA template sequence 3'-CAA*A*CCCAA*A*C-5' 47A-MT-hTer. Both the WT-hTER and 47A MT-hTer were cloned into the pHRCMVGFPWSin18 lentiviral vector under the IU1 promoter (see A in Additional file 14).

### Virus production, stable and transient expression of telomeric proteins and heterochromatin

Lentivirus was generated as described previously [45]. Briefly, 5 μg of pMD.G plasmid (virus envelope plasmid), 10 μg of pCMVDR8.91 (virus packaging plasmid), and 15 μg of the lentivector (GFP-TRF1, GFP-TRF2, lentiviral empty vector, WT-hTER, or 47A MT-hTer) were cotransfected into amphotropic 293T cells using the Fugene 6 transfection method (Roche, Indianapolis, IN, US). Virus was harvested 48 and 72 hours post-transfection and filtered through 0.45-μm filters. Virus titers were calculated in 293T cells by counting the number of GFP-expressing foci divided by the dilution factor (10^6^-10^7 ^transduction units/ml viral titer is achieved). For virus infection, culture cells were incubated with culture medium-diluted virus supernatant supplemented with 8 μg/ml of polybrene (Sigma, St Louis, MO, US) for 8 hours. A virus titer of 20 transduction unit/cells were used for a > 95% infection efficiency. GFP color starts to appear after 13 hours of infection and low expression monoclonal cell population stables were purified with hygromycin (200 μg/ml) (Invitrogen, Carlsbad, CA, US) or puromycin (2 μg/ml) (Sigma, St Louis, MO, US) selection for 5 days. Weak expression stables were chosen for Western analysis and imaging analysis. For cell growth measurement and cell cycle analysis, 2 × 10^4 ^cells were reseeded in triplicates in six-well plates 48 hours after virus infection, and the cell numbers were counted every day or cells harvested for propidium iodide (Molecular Probes, Carlsbad, CA, US) staining and fluorescence activated cell sorting analysis.

Clonal lines with the lowest average expression from Western blotting analysis were chosen for comparison with the endogenous level of TRF1 (60 to 65 kilobase pairs) or TRF2 (66 kilobase pairs) on 10% or 7.5% sodium dodecyl sulfate (SDS)-polyacrylamide gel electrophoresis gels for telomere dynamic analysis. For the low-expressing stable lines used in these analyses, the cell proliferation rates, morphology and cell cycle distribution (around 80% of the cells were in G1 in these cultures) were verified as showing no significant differences from the parental line (see A in Additional file 1). CFP-HP1α plasmid was transiently transfected into UMUC3 cells using Fugene 6 transfection and cells were imaged 36 hours later.

### Cell culture and drug treatments

Mammalian cancer cell lines were used in these analyses, as cell lines are known generally to retain the genomic characteristics of the primary tumor [46]. For all analyses, we used cell lines that do not contain PML bodies to observe systematically telomere motion in live cells. PML bodies are free nuclear particles that are not part of the chromosomes, and contain telomeric proteins and other telomeric components [27,28].

The endothelial bladder carcinoma cancer cell line UMUC3 was purchased from the American Type Culture Collection (ATCC), (#CRL-1749). We observed that the UMUC3 karyotype was stable over the course of this and other studies in our laboratory [39,47], consistent with the near-triploid karyotype and modal chromosome number of 80 described for this line by the supplier (ATCC). The HeLa cell line (cervical adenocarcinoma cancer cell line) was purchased from ATCC (#CCL-2). H1299, an epithelial lung carcinoma cancer cell line, was purchased from ATCC (#CRL-5803). Cells were cultured under standard cell culture conditions (37°C, 100% relative humidity, 5% CO_2_) in Dulbecco's modified Eagle's medium (Gibco, Langley, OK, US) supplemented with 10% fetal bovine serum (Gibco, Langley, OK, US), 2 mM L-glutamine (Gibco, Langley, OK, US), 100 U/ml penicillin and streptomycin (Gibco, Langley, OK, US). LOX cells (a human amelanotic melanoma cancer cell line) were cultured in RPMI medium (Gibco, Langley, OK, US) with the same supplements as above. Clonal cells expressing either GFP-TRF1 or GFP-TRF2 from each cancer cell line were generated by picking a single cell colony. HEPES (*N*-2-hydroxyethylpiperazone-n-2-ethanesulfonic acid) buffer (pH 7.4) was added to cell culture medium at a final concentration of 20 mM to avoid the need for carbon dioxide gas before imaging.

ATP depleting medium was made in culture medium, supplemented with 50 mM 2-deoxy-D-glucose (Sigma, St Louis, MO, US) and 6 mM sodium azide (Sigma, St Louis, MO, US). After washing away azide, cells were allowed to rest for 30 minutes before imaging again (azide wash). The methylating agent MMS (Sigma, St Louis, MO, US) was added at a concentration of 0.01% for 1 hour before imaging and immunofluorescence. WT-hTER lentivirus was used to infect the UMUC3 cells for 20 days, and analyzed for telomere length before imaging. 47A MT-hTer lentivirus was used to infect the cells for 3 to 6 days before imaging and immunofluorescence.

### Immunofluorescence and immunoblotting

For immunofluorescence, cells were fixed in chilled 4% paraformaldehyde, permeabilized with 0.5% NP-40 in phosphate buffered saline (PBS), blocked in 5% bovine serum albumin (BSA), incubated with the primary antibody in 5% BSA in PBS, washed, and then incubated with either Alexa fluor 568 or Alexa fluor 488-conjugated secondary antibody (either anti-mouse or anti-rabbit against the primary antibody) (Molecular Probes, Carlsbad, CA, US) in 5% BSA in PBS, washed, and finally in 0.15 μg/ml 4',6-diamidino-2-phenylindole (DAPI) before mounting. Indirect immunofluorescence showed the expected complete colocalization of telomeric signals from GFP-TRF1 with anti-TRF2 antibody or GFP-TRF2 with anti-hRap1 antibody (Figure 1C). For HP1α plasmid immunofluorescence, no DAPI staining was performed.

For immunoblotting, cells were lysed in radioimmunoprecipitation assay buffer (50 mM Tris/HCl (pH 7.4), 150 mM NaCl, 1% Triton X-100, 1% sodium deoxycholate and 0.1% SDS, supplemented with a complete protease inhibitor cocktail from Roche, Indianapolis, IN, US). Lysates were quantitated using Bradford assay (BioRad) and 15 μg of protein were mixed with SDS sample buffer (0.08 M Tris (pH 6.8), 2.0% SDS, 10% glycerol, 0.1 M dithiothreitol, 0.2% bromophenol blue) before boiling for 5 minutes and then subjected to immunoblotting with the antibodies.

### Antibodies

The following antibodies were used for indirect immunofluorescence analyses and Western blotting: mouse monoclonal antibodies TRF2 (#611200, BD, Franklin Lakes, NJ, US), β-actin (#A5441, Sigma, St Louis, MO, US), rabbit polyclonal antibodies 53BP1 (#A-300-273A-3, Bethyl, Montgomery, TX, US), ATM pS-1981 (#AF1655, R&D systems), Rap1 (#A300-306A, Bethyl, Montgomery, TX, US), γ-H2AX pS-139 (#NB100-384, Novus Biologicals, Littleton, CO, US) and TRF1 (#Ab1423, Abcam, Cambridge, MA, US).

### Telomere length analysis

A ^32^P-labeled telomeric (CCCTAA)_3 _probe was purified using the microsystem spin column (Bio-Rad) and hybridized to HinfI and RsaI-digested human genomic DNA at 37°C overnight. DNA samples were extracted from cells using a genomic DNA purification kit (Gentra, Minneapolis, MN, US) and separated on a 0.65% agarose gel. Southern results were exposed with a phosphor-imager.

### OMX microscopy

Cells were seeded on to a glass-bottomed cell culture dish (Bioptechs, Butler, PA, US) for OMX microscopy. This microscope is described in Schermelleh et al [48]. A ×60/1.25 water-immersion objective (Olympus) was used to collect 3-D stacks of 17 focal sections (an entire nucleus of 6–8 μm) with 0.5 μm focal step, once a second for 200 seconds. OMX requires 100 milliseconds (the fastest reported imaging speed to date) to acquire one complete 3-D live cell nucleus stack. Cells were exposed for 5 milliseconds to excitation light from a 500 mW 488 nm laser attenuated to 10% transmission with a neutral density filter. The total duration of timelapse imaging is limited by photobleaching (at the end of such data collection, fluorescence intensity is roughly half) (see D in Additional file 1). The high resolution 4-D data analysis allows the development of the system to accurately and statistically track any punctate chromatin foci including telomeres reported here.

### Image analysis

#### Track4D (4-D tracking)

Telomere tracking requires a robust image processing algorithm to localize the fluorescent spots in noise-limited minimal exposure images. We applied a 3-D Wiener filter with telomere modeled by a Gaussian with full-width half-height of 2.5 pixels. The filtered image was then subjected to a non-linear thresholding procedure based on intensity and local contrast threshold criteria for each pixel and its six 3-D neighbors. This algorithm reliably segmented telomere spots throughout the duration of the experiment despite bleaching.

3-D contiguous components analysis provided for each telomere a set of quantitative parameters, including local background, center of 'mass' (weighted by the background-subtracted fluorescence intensities), position of maximum intensity, volume, the number of boundary pixels, and the background-subtracted integrated intensity. Due to the high and inhomogeneous background, the local background was determined from repeated dilation of each telomere segment to define the asymptotic value for the averaged intensity out of the segments as the background value. The parameters are then used to reject non-telomere spots (volume, intensity and surface-to-volume outliers) and time-track telomere motion. For dense time-sampling, tracking is simply achieved by searching for the closest telomere spot in the preceding and following time points.

We corrected for nucleus drift or rotation during the live imaging, which might have confounded interpreting telomere mobility (see E in Additional file 1).

For each telomere track (each line in the Track4D plot tracks an individual telomere, but colors of telomere tracks are random between plots), we calculated the 3-D distance to position at time zero (E2E) and the accumulated root-mean-square distance between consecutive times. The effective diffusion coefficient *D *was calculated from E2E using Einstein's diffusion equation, E2E = √(6*DT*) (*T *is the time in seconds). *D *is based on E2E distances > 0.3 μm, and sampling at 150 nm steps (1/6 of the microscope resolution in *z*).

#### TrackIt4D (tracks analysis)

The TrackIt4D (tracks analysis) program calculated the accumulated path distance (PATH). It also generates a histogram plot for the *D *values of each single nucleus. An accumulated histogram was generated based on around 400 telomeres or heterochromatin spots. The distance that a cell nucleus has drifted during imaging is indicated by a line near the bottom of the *x*-axis (timescale). Drifts are corrected when quantifying telomere motion.

Average E2E was calculated independently. Telomere foci were first sampled above the background noise using the custom-written Python software and telomere mobility was tracked, calculated (Additional file 9) and plotted. By averaging E2E for all pairs with a given time difference, there is less noise. Briefly, telomere motion statistics were calculated from average E2E distance over sub-intervals. The rate of timelapse imaging was 1 second in all cases. All distances traveled by the same telomere over time intervals of the same length were averaged together and plotted. Telomeres tracked for *N *seconds were plotted for intervals up to *N*/2 seconds (*N *is any number). Global nucleus motion was estimated from the drift in the average spots position and subtracted from all spots. Rotational motion was found negligible for the tracking times used.

Trajectory images were created and plotted on top of original nucleus images after TrackIT4D tracking. The association of each telomere to the nuclear periphery was approximately assessed by viewing the nucleus as an ellipsoid and using the Mahalanobis distance from the center to define proximity to the nuclear periphery. Nuclear volumes were estimated by drawing the nuclear periphery contour with polygons and evaluation of the area and volume of the nucleus in all optical sections using the Priism software suite.

### Statistical analysis

Error was estimated by statistical bootstrapping. Bootstrapping of histograms of *D *values for non-parametric statistical comparison was done by repeatedly and randomly splitting the histograms of 400 telomeres into two sub-groups of 200 telomeres each, and averaging mean and standard deviation for the mode or the Komolgorov-Smirnov (K-S) comparison test between them. Diffusion coefficient histograms were accumulated for about 20 nuclei (>400 telomere tracks) for each experimental condition. For skewed, certainly non-normal distributions, K-S statistics have to be used. The difference between histograms was scored by the K-S comparison test. Error bars were estimated from statistical bootstrap. A K-S score of more than three times the error (standard deviations) from bootstrapping procedures is considered significant.

## List of abbreviations

3-D: three-dimensional; 4-D: four-dimensional; ALT: alternative lengthening of telomeres; ATCC: American Type Culture Collection; BSA: bovine serum albumin; DAPI: 4',6-diamidino-2-phenylindole; E2E: end-to-end; GFP: green fluorescent protein; K-S: Komolgorov-Smirnov; MMS: methyl methanesulfonate; MSD: mean-square-distance; OMX: optical microscope platform; PBS: phosphate buffered saline; PML: promyelocytic leukaemia; SDS: sodium dodecyl sulfate.

## Competing interests

The authors declare that there are no competing interests.

## Authors' contributions

XYW constructed the fusion telomere protein-fluorescent protein gene constructs, performed cellular transformations and cell cultures, image collection and analyses and participated in drafting and writing the manuscript. PMC designed the analytical approaches, performed image acquisition and image analyses and participated in writing the manuscript. ZK conceived the analytical methods, wrote the software used for telomere motion analyses and participated in writing the manuscript. LX constructed the fusion telomere protein-fluorescent protein gene constructs, trained XYW in the cellular systems and some of the methods used in this study, and participated in the interpretation of results. JWS conceived and designed the OMX microscope and high time-resolution approach and participated in writing the manuscript. EHB conceived the biological study and experimental designs, interpreted the results, and participated in writing the manuscript. All the authors read and approved the manuscript.

## Supplementary Material

Additional file 1 **Visualization of telomeres in live cells. (A) Cell cycle distribution of clonal cultures.** Around 80% of the control parental cells are in G1 in these unsynchronized cultures. Clones expressing the green fluorescent protein (GFP)-TRF1 or GFP-TRF2 construct at low levels (GFP-TRF1 Clone 5 and GFP-TRF2 Clone 2) were chosen for use in the analyses in this work and were verified not to be different from those of the parental line in their cell cycle profile. (B) The intensity distribution of telomeres within a single representative nucleus of UMUC3 (upper right corner) shows a broad range. (C) One complete three-dimensional image stack of 17 *z*-sections was acquired every second (every third section shown). Green circles mark the telomeres tracked at that particular *z*-section. (D) Photobleaching graph of the image taking telomere intensity versus time shows a smooth reduction factor of two occurred during 200 seconds of imaging due to photobleaching. (E) Examples of nuclear trajectory images corrected for nuclear drift. Click here for file

Additional file 2 **Visualization of telomeres in UMUC3 mammalian cancer cells using OMX live imaging.** The movie was recorded 10 frames/second. Around 40 to 60 telomeres were accurately located and tracked. The movie also shows a large variation of telomere intensities within a single nucleus. Click here for file

Additional file 3 **Visualization of telomeres in UMUC3 mammalian cancer cells using OMX live imaging, showing the large variability in telomere motion within a single nucleus.** The movie was recorded 10 frames/second. Three UMUC3 nuclei were shown, the weaker expression cells were later chosen as clonal cell lines for minimal perturbation of telomeres during dynamic analysis. Click here for file

Additional file 4 **Visualization of telomeres in UMUC3 mammalian cancer cells using OMX live imaging, showing the large variability in telomere motion within a single nucleus.** The movie is an enhanced-brightness picture of the bottom UMUC3 nucleus from Additional file 3. Visual inspection revealed heterogeneous telomeric motion within a single live cancer cell nucleus: some were moving rapidly, while others were moving at a slower speed. Click here for file

Additional file 5 **An example of visualization of telomere motion showing nuclear drift during image taking, using OMX live imaging.** The movie was recorded 10 frames/second. Such nuclear drift was corrected for in the analyses. Click here for file

Additional file 6 **Six different ways of visualizing and quantifying telomere motions in live cells.** (A) Kymographs (vertical axis is 10 frames/second), showing individual telomeres as tracks projected on to a two-dimensional image (shown) or as three-dimensional (3-D) (see Additional files 7 and 8). (B) A plot of individual telomere tracks showing the movement of each individual telomere as its projected position in the *xy*-plane (visualized as the horizontal plane) as a function of time *T *(vertical axis); this plot does not depict the changes with time in the position of the telomere in the *z*-axis, although the data were acquired. (C) A projection of the trajectory of each telomere showing its position in 3-D space as a function of time for 200 consecutive seconds; each green dot shows the distance path of the telomere traveled in 200 seconds. (D) For each telomere at time *T*, the end-to-end (E2E) distance in 3-D space the telomere has traveled from its original starting point position at time 0 (each line in the plot tracks the distance against time for an individual telomere, but the colors of the telomeres tracked are random). (E) The cumulative path distance traveled by a telomere between time 0 and time *T *200 seconds. The line near the bottom of the *x*-axis indicates the distance a cell nucleus has drifted during imaging, which is corrected when quantifying telomere motion. (F) The average E2E distances for each telomere track. The E2E distances at all pairs of time points *T *seconds apart were averaged (see Additional file 9). Datasets using (C) to (E) are corrected for any nucleus drift during imaging. Click here for file

Additional file 7 **Three-dimensional visualization of telomere motion using kymographs.** The movie was recorded 10 frames/second. Rotational movie of kymographs showing telomere motion in one representative UMUC3 cell nucleus. This allows 360 round inspection of the telomere motion in three-dimensional (3-D) space at any time point. Click here for file

Additional file 8 **Three-dimensional visualization of telomere motion using kymographs.** The movie was recorded 10 frames/second. Telomere dynamics are visualized in kymographs as lines in 3-D space. Kymographs of three UMUC3 cell nuclei from Additional file 3 were shown. Click here for file

Additional file 9 **Quantifying telomere motions in live cells.** Averaging of end-to-end (E2E) distances for quantitative measurement of telomere motion. The path of a moving particle (bottom left) is divided up into intervals of integer numbers of 1-second time points. For each 1-second increase in the interval considered, the number of intervals decreases by one; thus larger intervals have fewer samples and are more subject to stochastic variation. Lengths traveled during each interval are shown to the right of each figure. These lengths are averaged together (average length shown under the black line). When the average lengths from a particle undergoing unconstrained random diffusion are plotted, they scale with the square root of elapsed time. This procedure helps to reduce measurement noise, especially for short time differences. Telomeres tracked for *N *seconds are plotted for intervals up to *N*/2 seconds. Click here for file

Additional file 10 **Heterogeneous motions of telomeres.** (A) An additional six other representative tracks of telomeres in UMUC3 nuclei, each line is an end-to-end (E2E) track of a single telomere. The top three panels show telomeres tracked using green fluorescent protein (GFP)-TRF1 and the bottom three panels show telomeres tracked using GFP-TRF2. These E2E measurements were used to calculate the effective diffusion coefficient *D *using Einstein's diffusion equation, E2E = √(6*DT*) (where *T *is the time in seconds). (B) Bootstrapping procedure to estimate errors in comparing histograms of *D *values. The procedure involves repeatedly and randomly splitting two populations of 200 telomeres each, and averaging the mean and standard deviation for the mode. Figure 2E was taken as a reference. One such split is shown in the right two panels. (C) Log-scale histogram showing that the skewed histogram for effective diffusion coefficient *D *values does not present a log-normal distribution. Click here for file

Additional file 11 **The wide range of heterogeneity of motion is telomeric specific.** (A) Cumulative path distance measurements of telomeres and heterochromatin spots. TrackIt4D analysis of heterochromatin motion in three-dimensional space shows that the heterochromatin moves at much less motion compared with telomeres. *D *values for UMUC3 cell heterochromatin loci in our experimental settings are consistent with reports on heterochromatin regions in yeast, *Drosophila *and mammalian models (*D *values of 0.05–1.3 × 10^-4 ^mm^2^/second). (B) Overlap of *D *histograms for telomeres versus heterochromatin spots. The Komolgorov-Smirnov comparison value for these two histograms is 0.517 ± 0.029, which is statistically significant (*N *= around 400 telomeres, *N *= around 400 heterochromatin spots). Click here for file

Additional file 12 **Telomere movement is energy dependent.** (A) Three-dimensional trajectory images of telomeres show dampening of telomere motion upon azide treatment. Medium containing 6 mM sodium azide was perfused into the live cell chamber for 30 minutes and images were acquired before, and after perfusion, and after azide wash out (upper three panels). This condition is similar to that used to show energy dependence of anaphase chromosome movement [29,49]. Washing away azide restores the telomere motion. These findings were corroborated by analyses of plottings of distance in space that at the accumulated telomere movements path as a function of time *T *seconds, telomeres had moved away from their original position at time 0 second after azide wash (lower panels and Figure 4A). (B) Nuclear areas/volumes were estimated by drawing the nuclear periphery contours with polygons in all optical sections and evaluation of the area by adding the polygons to estimate the area/volume of the nucleus. These polygon measurements of cell nuclei showed a <10% change in either area or volume of cells under the experimental perturbations due to azide treatment. Hence telomeres before, during and after the azide treatment had the same total space potentially available for sampling as the controls ruling out nucleus compaction. (C) Telomere histograms of control nuclei versus azide-treated cells (*N *= around 400 telomeres). Notably, the peak position mode for the histograms of *D *values did not change; rather, the faster moving telomere population was selectively diminished compared with the control. Click here for file

Additional file 13 **Telomere movements are related to telomere shortness and can be altered experimentally.** (A) Telomere motion is lower in cell lines with longer telomeres. Track4D tracings are shown of telomere tracks in different cancer cell lines, whose mean telomere lengths (kilobases) are known to differ. Datasets of images were obtained using cells (from a single clone) expressing low levels of fluorescently tagged TRF1, as described for the UMUC3 cells, to track telomere dynamics. Mean telomere lengths by Southern blotting for the cell lines analyzed are: LOX, >30 kilobases; H1299, mean 12 kilobases; HeLa, mean 5 kilobases; UMUC3, 2–5 kilobases. (B) Quantitative analysis of datasets for the four cell lines for which representative plots were shown in (A) bold. *N *= around 200 telomeres for each cell line. (C) Track4D program allows picking up of an individual telomere from all tracked telomeres (indicated by the number at the side of the *y*-axis) in the nucleus. The intensity value of the particular telomere was then matched with its effective diffusion coefficient *D*. (D) Matching the group of telomeres having the highest and lowest 20% in intensity of the individual telomere with top and bottom 20% effective diffusion coefficient *D *in the same cell nucleus reveals that the majority of the fast moving telomeres are contributed by the shorter telomeres within the same nucleus (*N *= around 400 telomeres). (E-G) Experimentally increasing the mean bulk telomere length causes decreased telomere movements. The average telomere length was extended by expressing extra WT-hTER in the same cell background (UMUC3 cell line). (E) Cumulative distances paths traveled by telomeres in two representative UMUC3 cell nuclei with unperturbed (left panel, empty vector control) or lengthened (right panel) telomeres. (F) Telomere *D *value histograms of control (lentivector)-treated nuclei versus the WT-hTER overexpressing cells (*N *= around 400 telomeres). The Komolgorov-Smirnov (K-S) non-parametric comparison between parental UMUC3 cells and lentivector-treated cells scored 0.013 ± 0.031, which is significantly indifferent. However, the K-S comparison between cells expressing lentivector versus the WT-hTER histograms gave a score of 0.199 ± 0.027, which is significantly different (see Additional file 18). Hence telomere motion is significantly slower in cells in which the bulk telomere length was increased experimentally in the same cell background. (G) Intensity changes 1–2-fold in cell nuclei of UMUC3 cells expressing extra WT-hTER at a level 3–5-fold above endogenous levels (*N *= around 200 telomeres). By thus experimentally increasing average telomere length, these results also independently confirmed that the telomeric fluorescence signals are indeed telomeric, because their average intensity also increased; when telomeres in UMUC3 cells are extended with WT-hTER, the mean intensity value shifts to a higher value (right panel). This supported the assumption of using intensity values as a relative measure of telomere lengths. Click here for file

Additional file 14 **Telomere movement could be altered experimentally.** (A) A schematic diagram of the mutant-template telomerase RNA MT-hTer expression lentivector (left panel), and the sequences of the 47A MT-hTer template and the telomeric repeats it causes to be incorporated (right panel) [38,50]. (B) Three-dimensional (3-D) kymographs of telomere motion projected on to a two-dimensional plane. 47A MT-hTer produces high motility and rates of movement of some telomeres, as seen by the group of wavy lines to the right of the kymograph for this representative cell nucleus. (C) 3-D trajectory images of telomeres showing greater motion in cells that had expressed 47A MT-hTer for 6 days. (D) Cumulative path distances traveled by telomeres under two different damaging conditions. 47A MT-hTer induces greater telomere motion, but methyl methanesulfonate (MMS), a general DNA damaging drug, does not. To test whether nuclear expansion or shrinkage is due to the 47A-expression or could have contributed to the changes in telomere motility, nuclear volumes were estimated by drawing the contour with polygons and evaluation of the area and adding the area of all optical sections. These measurements confirmed that any volume change was <5% upon 47A MT-hTer or MMS treatments to the cells. Hence, telomere motion is greatly increased when telomeres are uncapped by MT-hTer 47A, but not by general DNA damage (MMS). (E) Colocalization of telomeres with DNA damage response foci visualized by staining with an antibody against 53BP-1, a DNA damage protein (left three panels). In UMUC3 cells, introduction of a 47A MT-hTer construct induced a rapid DNA damage response and telomere uncapping response, including telomere dysfunction-induced foci, within 3 days. The 47A MT-hTer expressing cells showed more than 70% of DNA damage foci colocalization on telomeres, indicative of telomere-specific damage (telomere uncapping) (the bar plot in the right panel). Similar results were shown for colocalization of telomeres with other DNA damage proteins: ATM pS-1981 and γ-H2AX pS-139 upon 47A MT-hTer expression. In contrast, the 0.01% of MMS treatment for 1 hour induced DNA damage foci that did not colocalize with telomeres (left three panels shows the immunofluorescence pictures and the right panel shows the quantitative bar plot). (F) Histograms of telomere motion *D *values for control (empty lentivector-treated) nuclei versus 47A MT-hTer expressing cells (*N *= around 400 telomeres). Komolgorov-Smirnov non-parametric comparison between the two histograms was performed. The score was 0.269 ± 0.025, indicating a highly significant difference in the telomere motion between the controls versus the cells with uncapped telomeres. Click here for file

Additional file 15 **Sizes and diffusion coefficients of particles in the nucleus and of chromatin loci.** (A) Copied from Gorisch SM, Lichter P, Rippe K. (2005): Dextran particles in different milieus. (B) Various other measurements of D in the nucleus. Click here for file

Additional file 16 **Justified assessment of quantifying telomere motions by effective diffusion coefficients.** (A) Averaged mean-square-distance (MSD) versus time for around 800 telomeres shows that telomere motion imaged within 200 seconds does not reach the plateau indicative of constrained motion. The plot shows the MSD curves for the telomeres and demonstrates that the MSD value does not decrease (saturate the volume of nuclear three-dimensional (3-D) space available for movement) over a 200-second time interval of observation. (B) A 3-D trajectory image at any *z*-section and any time of a UMUC3 nucleus shown to estimate the distance of each telomere to the nuclear periphery (indicated by the red lines). Click here for file

Additional file 17 **Green fluorescent protein (GFP)-TRF1 labeled, parental UMUC3 clonal cells were imaged using OMX microscopy.** Cells with similar intensity/AU under the same light exposure were chosen and their telomere motility (*N *= around 200) analyzed. The end-to-end (E2E) measurements of these telomeres were used to calculate their effective diffusion coefficients *D *(10^-4 ^μm^2^/second) using Einstein's diffusion equation, E2E = √(6*DT*) (where *T *is the time in seconds). Each telomere's intensity was plotted against its *D *value to give a scatter plot indicative of the relationship between telomere length and motility. The linear regression line shows a value of *R*^2 ^= 0.5917 in the graph. Click here for file

Additional file 18 **Table 1 – A summary of the Komolgorov-Smirnov non-parametric comparison between histograms discussed in the text.** A Komolgorov-Smirnov (K-S) score three times the error (standard deviation) is considered significant. Red scores indicate statistically significant different histograms, while the orange score is at the borderline for statistically significant difference between the two histograms under comparison. The first value in the right column is the K-S value between histograms, and the second value after ± is the bootstrap value (error) for each histograms. Hence, the table shows that there is significant difference when comparing histograms of parental or vector control cells with either heterochromatin, azide-treated cells, WT-hTER-treated cells, or 47A MT-hTer-treated cells (red-colored scores and orange score). Bootstrap analysis for all histograms within error bars of < 0.05, hence comparisons between histograms are valid. Click here for file
