# Supplementary figures and images for: Rapid telomere motions in live human cells analyzed by highly time-resolved microscopy
Source: Epigenetics Chromatin. 2008 Oct 27;1:4. doi: 10.1186/1756-8935-1-4 (PMC2585561; doi:10.1186/1756-8935-1-4)

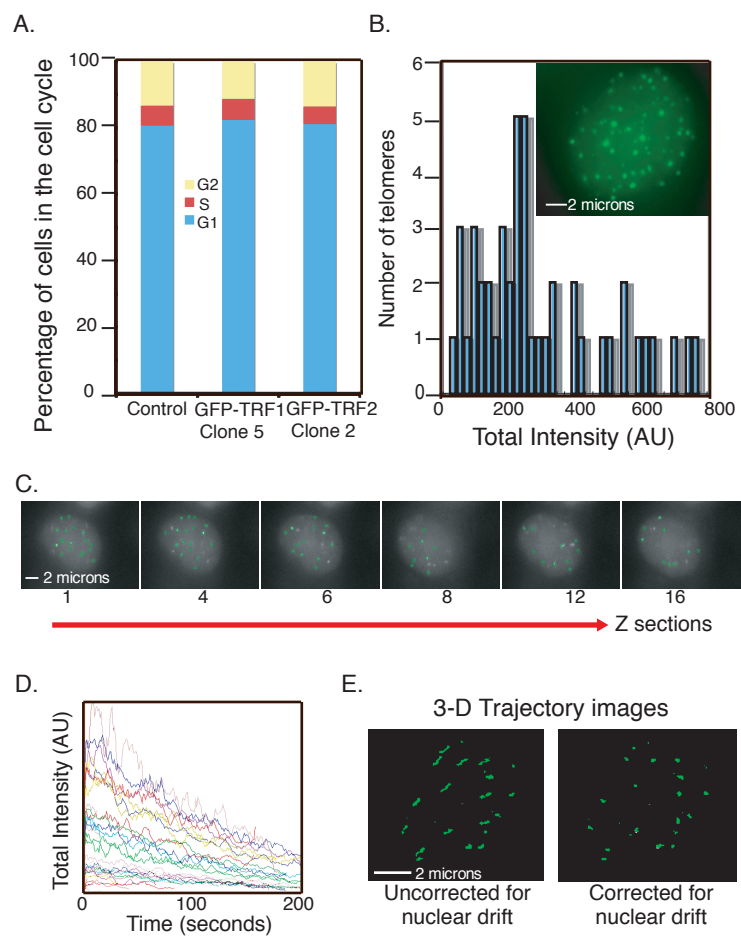

fig. S1

Supplement: Additional file 1 — Visualization of telomeres in live cells. (A) Cell cycle distribution of clonal cultures. Around 80% of the control parental cells are in G1 in these unsynchronized cultures. Clones expressing the green fluorescent protein (GFP)-TRF1 or GFP-TRF2 construct at low levels (GFP-TRF1 Clone 5 and GFP-TRF2 Clone 2) were chosen for use in the analyses in this work and were verified not to be different from those of the parental line in their cell cycle profile. (B) The intensity distribution of telomeres within a single representative nucleus of UMUC3 (upper right corner) shows a broad range. (C) One complete three-dimensional image stack of 17 z-sections was acquired every second (every third section shown). Green circles mark the telomeres tracked at that particular z-section. (D) Photobleaching graph of the image taking telomere intensity versus time shows a smooth reduction factor of two occurred during 200 seconds of imaging due to photobleaching. (E) Examples of nuclear trajectory images corrected for nuclear drift. [file 1756-8935-1-4-S1.pdf]

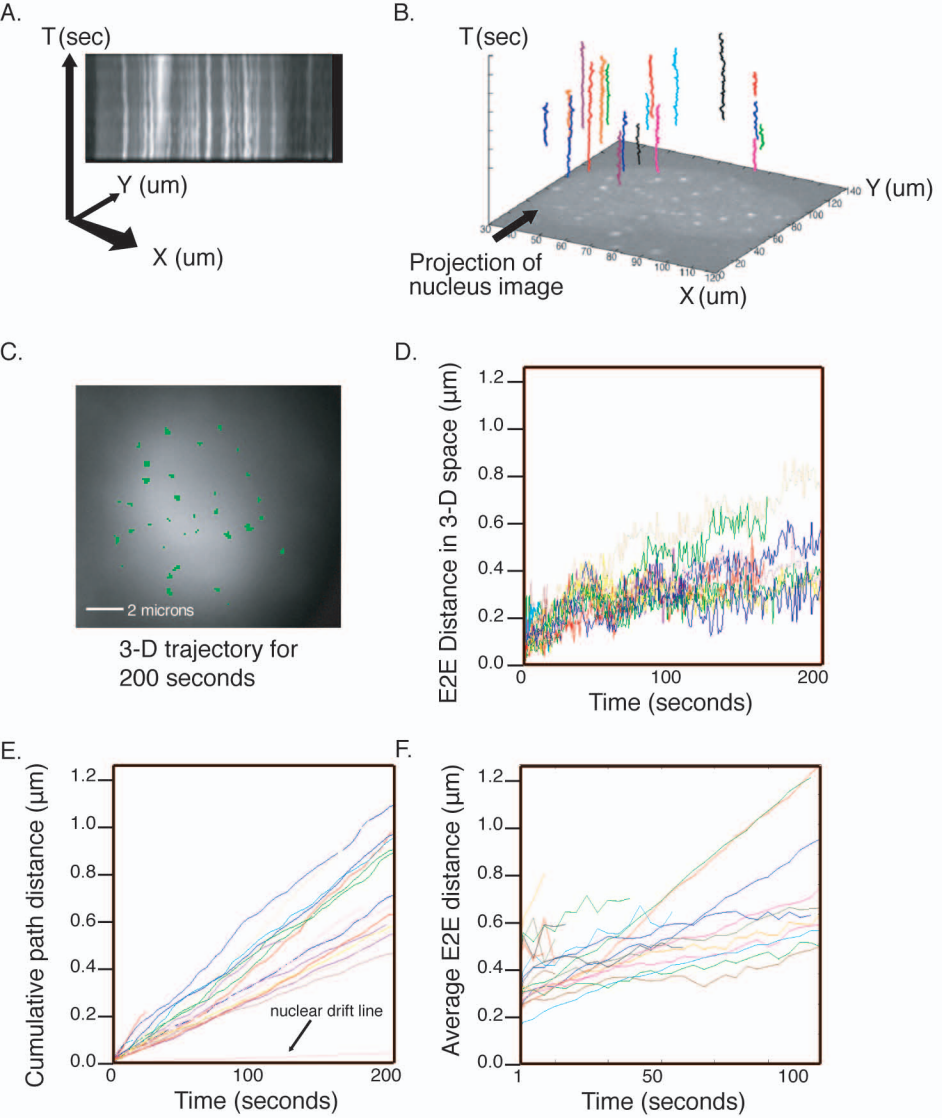

fig. S2

Supplement: Additional file 6 — Six different ways of visualizing and quantifying telomere motions in live cells. (A) Kymographs (vertical axis is 10 frames/second), showing individual telomeres as tracks projected on to a two-dimensional image (shown) or as three-dimensional (3-D) (see Additional files 7 and 8). (B) A plot of individual telomere tracks showing the movement of each individual telomere as its projected position in the xy-plane (visualized as the horizontal plane) as a function of time T (vertical axis); this plot does not depict the changes with time in the position of the telomere in the z-axis, although the data were acquired. (C) A projection of the trajectory of each telomere showing its position in 3-D space as a function of time for 200 consecutive seconds; each green dot shows the distance path of the telomere traveled in 200 seconds. (D) For each telomere at time T, the end-to-end (E2E) distance in 3-D space the telomere has traveled from its original starting point position at time 0 (each line in the plot tracks the distance against time for an individual telomere, but the colors of the telomeres tracked are random). (E) The cumulative path distance traveled by a telomere between time 0 and time T 200 seconds. The line near the bottom of the x-axis indicates the distance a cell nucleus has drifted during imaging, which is corrected when quantifying telomere motion. (F) The average E2E distances for each telomere track. The E2E distances at all pairs of time points T seconds apart were averaged (see Additional file 9). Datasets using (C) to (E) are corrected for any nucleus drift during imaging. [file 1756-8935-1-4-S6.pdf]

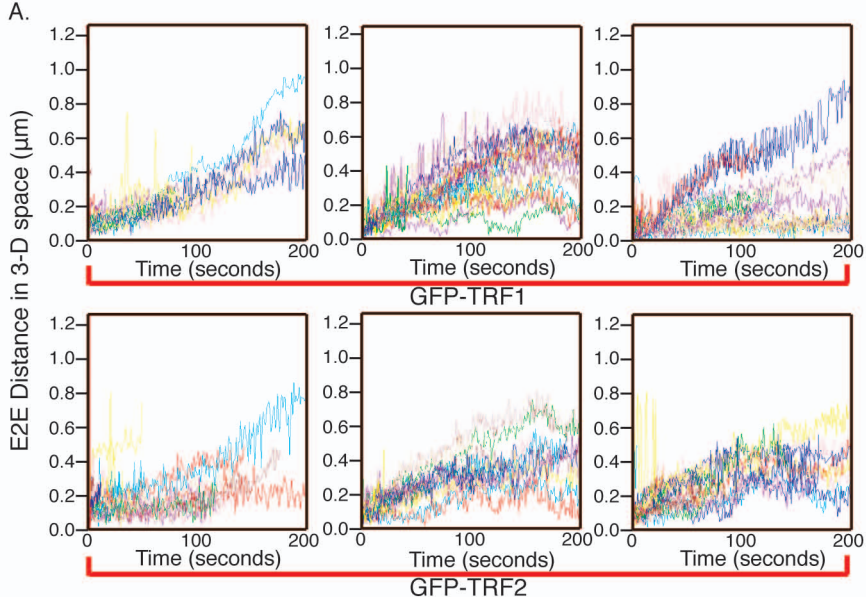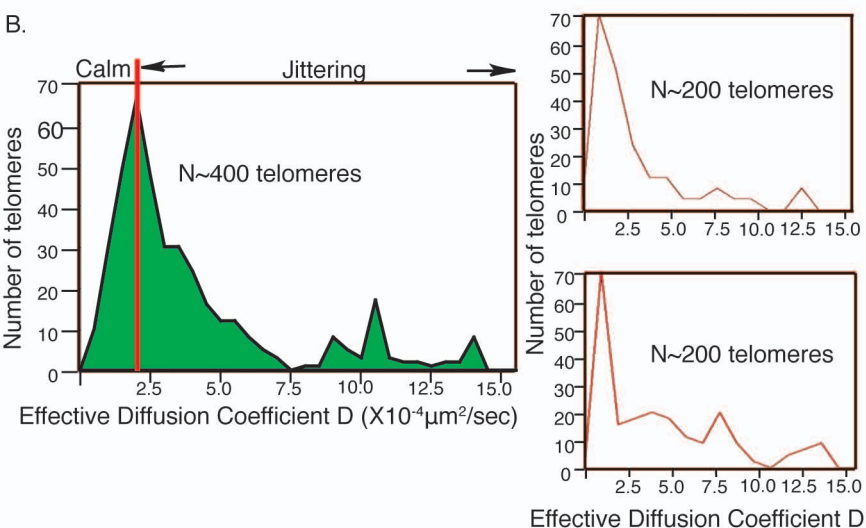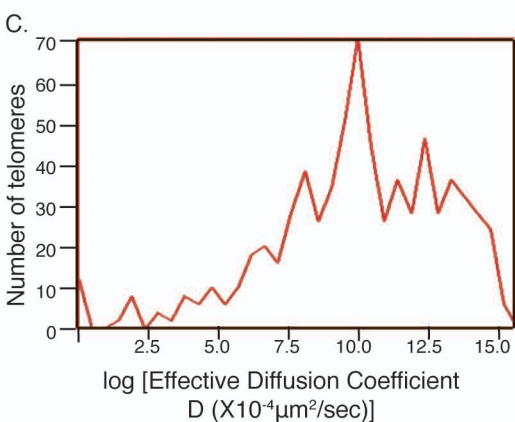

fig. S4

Supplement: Additional file 10 — Heterogeneous motions of telomeres. (A) An additional six other representative tracks of telomeres in UMUC3 nuclei, each line is an end-to-end (E2E) track of a single telomere. The top three panels show telomeres tracked using green fluorescent protein (GFP)-TRF1 and the bottom three panels show telomeres tracked using GFP-TRF2. These E2E measurements were used to calculate the effective diffusion coefficient D using Einstein's diffusion equation, E2E = √(6DT) (where T is the time in seconds). (B) Bootstrapping procedure to estimate errors in comparing histograms of D values. The procedure involves repeatedly and randomly splitting two populations of 200 telomeres each, and averaging the mean and standard deviation for the mode. Figure 2E was taken as a reference. One such split is shown in the right two panels. (C) Log-scale histogram showing that the skewed histogram for effective diffusion coefficient D values does not present a log-normal distribution. [file 1756-8935-1-4-S10.pdf]

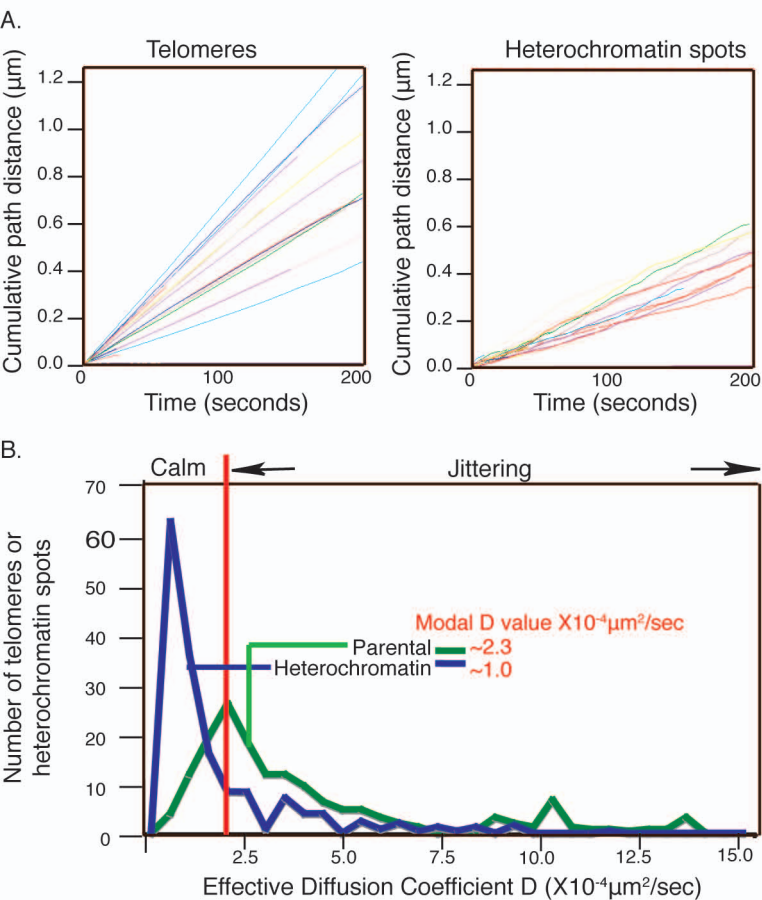

fig. S5

Supplement: Additional file 11 — The wide range of heterogeneity of motion is telomeric specific. (A) Cumulative path distance measurements of telomeres and heterochromatin spots. TrackIt4D analysis of heterochromatin motion in three-dimensional space shows that the heterochromatin moves at much less motion compared with telomeres. D values for UMUC3 cell heterochromatin loci in our experimental settings are consistent with reports on heterochromatin regions in yeast, Drosophila and mammalian models (D values of 0.05–1.3 × 10-4 mm2/second). (B) Overlap of D histograms for telomeres versus heterochromatin spots. The Komolgorov-Smirnov comparison value for these two histograms is 0.517 ± 0.029, which is statistically significant (N = around 400 telomeres, N = around 400 heterochromatin spots). [file 1756-8935-1-4-S11.pdf]

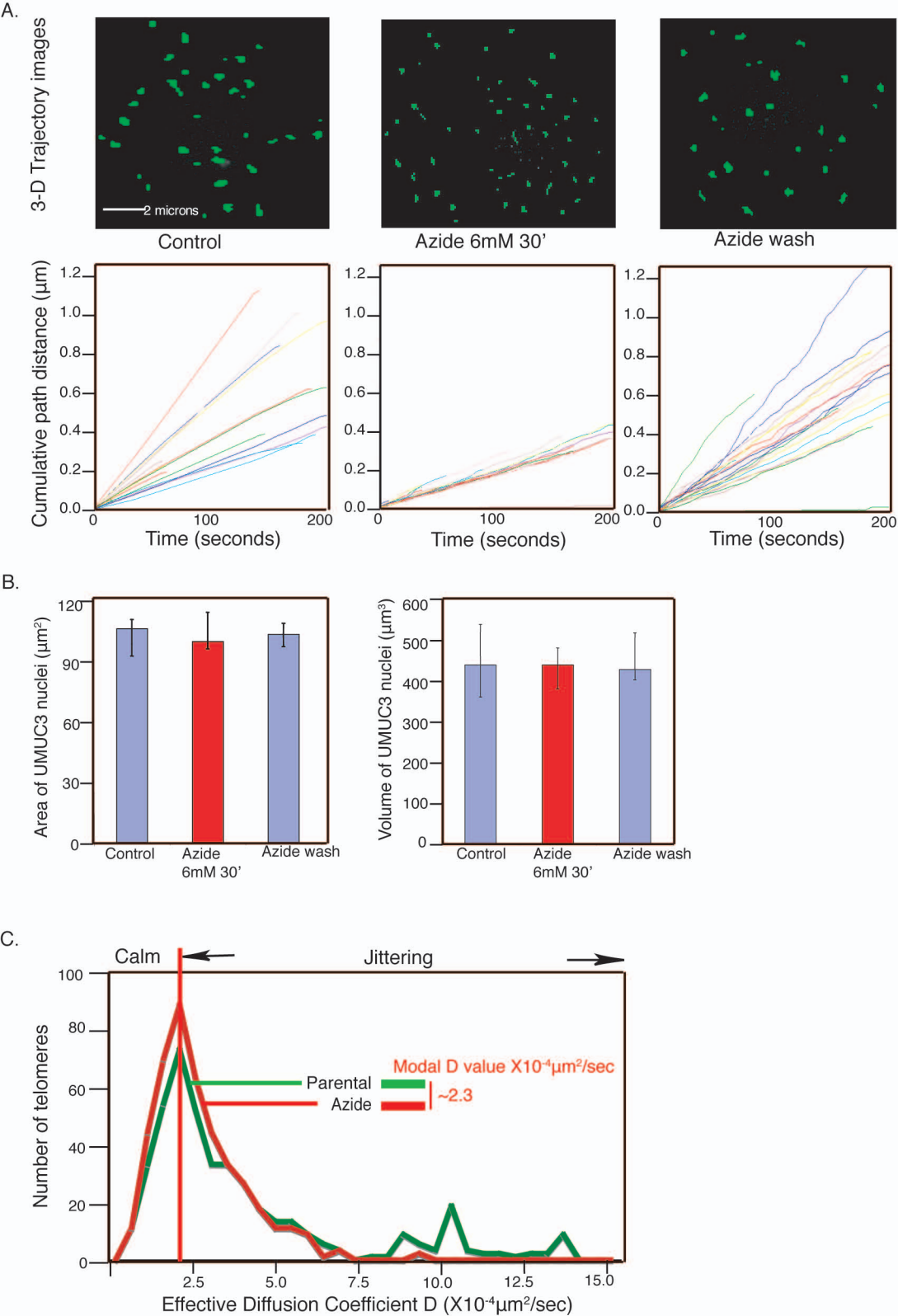

Supplement: Additional file 12 — Telomere movement is energy dependent. (A) Three-dimensional trajectory images of telomeres show dampening of telomere motion upon azide treatment. Medium containing 6 mM sodium azide was perfused into the live cell chamber for 30 minutes and images were acquired before, and after perfusion, and after azide wash out (upper three panels). This condition is similar to that used to show energy dependence of anaphase chromosome movement [29,49]. Washing away azide restores the telomere motion. These findings were corroborated by analyses of plottings of distance in space that at the accumulated telomere movements path as a function of time T seconds, telomeres had moved away from their original position at time 0 second after azide wash (lower panels and Figure 4A). (B) Nuclear areas/volumes were estimated by drawing the nuclear periphery contours with polygons in all optical sections and evaluation of the area by adding the polygons to estimate the area/volume of the nucleus. These polygon measurements of cell nuclei showed a <10% change in either area or volume of cells under the experimental perturbations due to azide treatment. Hence telomeres before, during and after the azide treatment had the same total space potentially available for sampling as the controls ruling out nucleus compaction. (C) Telomere histograms of control nuclei versus azide-treated cells (N = around 400 telomeres). Notably, the peak position mode for the histograms of D values did not change; rather, the faster moving telomere population was selectively diminished compared with the control. [file 1756-8935-1-4-S12.pdf]

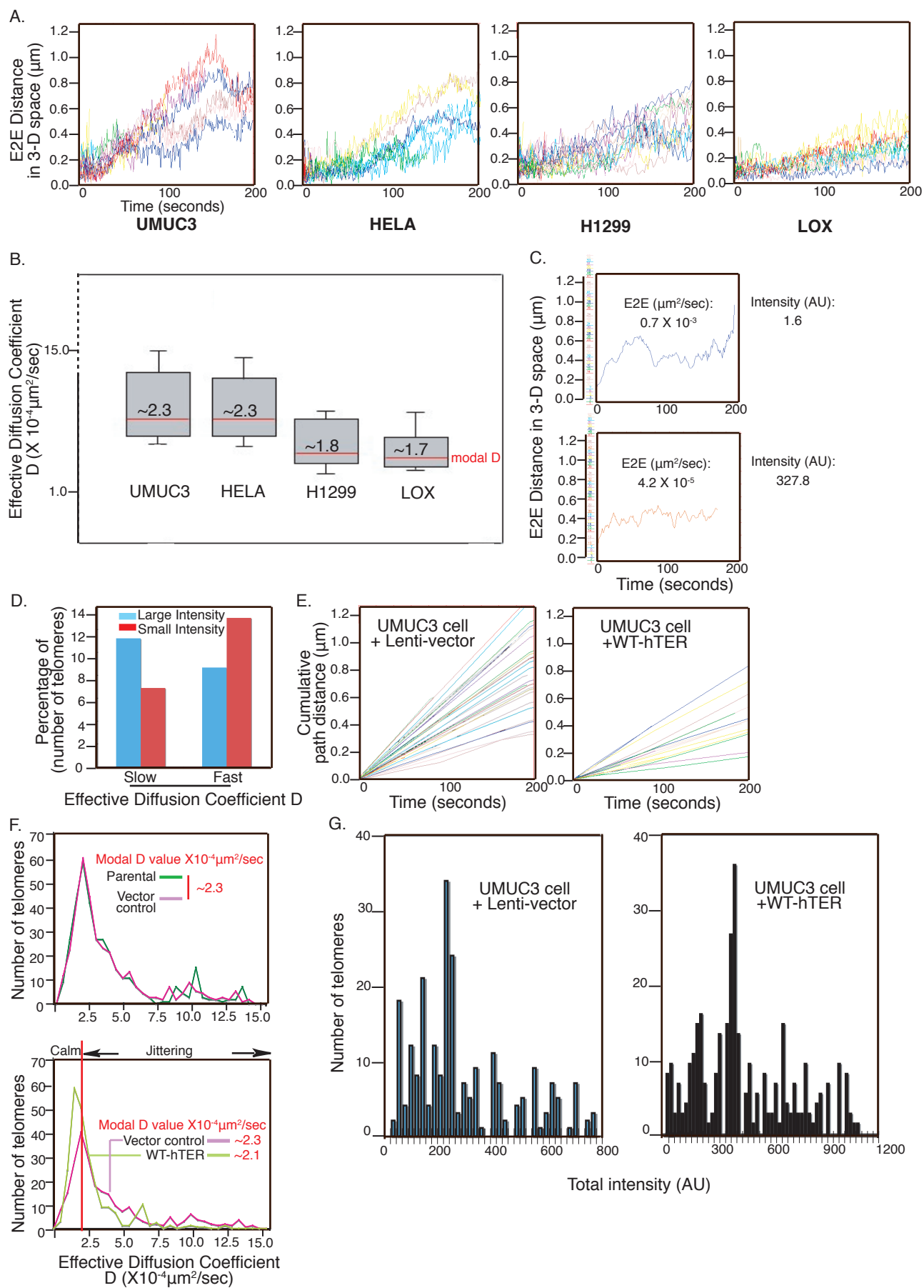

fig. S8

Supplement: Additional file 13 — Telomere movements are related to telomere shortness and can be altered experimentally. (A) Telomere motion is lower in cell lines with longer telomeres. Track4D tracings are shown of telomere tracks in different cancer cell lines, whose mean telomere lengths (kilobases) are known to differ. Datasets of images were obtained using cells (from a single clone) expressing low levels of fluorescently tagged TRF1, as described for the UMUC3 cells, to track telomere dynamics. Mean telomere lengths by Southern blotting for the cell lines analyzed are: LOX, >30 kilobases; H1299, mean 12 kilobases; HeLa, mean 5 kilobases; UMUC3, 2–5 kilobases. (B) Quantitative analysis of datasets for the four cell lines for which representative plots were shown in (A) bold. N = around 200 telomeres for each cell line. (C) Track4D program allows picking up of an individual telomere from all tracked telomeres (indicated by the number at the side of the y-axis) in the nucleus. The intensity value of the particular telomere was then matched with its effective diffusion coefficient D. (D) Matching the group of telomeres having the highest and lowest 20% in intensity of the individual telomere with top and bottom 20% effective diffusion coefficient D in the same cell nucleus reveals that the majority of the fast moving telomeres are contributed by the shorter telomeres within the same nucleus (N = around 400 telomeres). (E-G) Experimentally increasing the mean bulk telomere length causes decreased telomere movements. The average telomere length was extended by expressing extra WT-hTER in the same cell background (UMUC3 cell line). (E) Cumulative distances paths traveled by telomeres in two representative UMUC3 cell nuclei with unperturbed (left panel, empty vector control) or lengthened (right panel) telomeres. (F) Telomere D value histograms of control (lentivector)-treated nuclei versus the WT-hTER overexpressing cells (N = around 400 telomeres). The Komolgorov-Smirnov (K-S) non-parametric compa [file 1756-8935-1-4-S13.pdf]

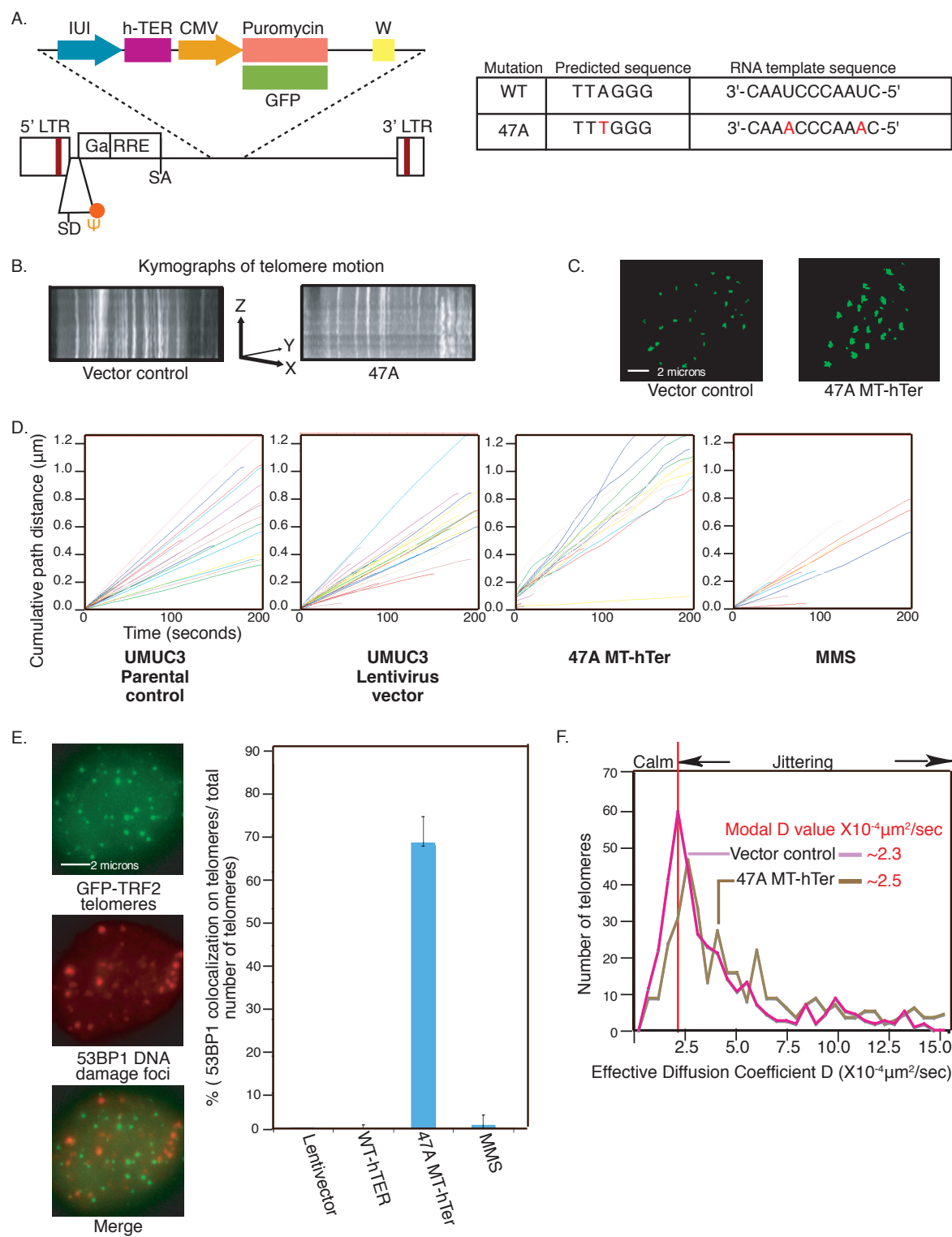

fig. S9

Supplement: Additional file 14 — Telomere movement could be altered experimentally. (A) A schematic diagram of the mutant-template telomerase RNA MT-hTer expression lentivector (left panel), and the sequences of the 47A MT-hTer template and the telomeric repeats it causes to be incorporated (right panel) [38,50]. (B) Three-dimensional (3-D) kymographs of telomere motion projected on to a two-dimensional plane. 47A MT-hTer produces high motility and rates of movement of some telomeres, as seen by the group of wavy lines to the right of the kymograph for this representative cell nucleus. (C) 3-D trajectory images of telomeres showing greater motion in cells that had expressed 47A MT-hTer for 6 days. (D) Cumulative path distances traveled by telomeres under two different damaging conditions. 47A MT-hTer induces greater telomere motion, but methyl methanesulfonate (MMS), a general DNA damaging drug, does not. To test whether nuclear expansion or shrinkage is due to the 47A-expression or could have contributed to the changes in telomere motility, nuclear volumes were estimated by drawing the contour with polygons and evaluation of the area and adding the area of all optical sections. These measurements confirmed that any volume change was <5% upon 47A MT-hTer or MMS treatments to the cells. Hence, telomere motion is greatly increased when telomeres are uncapped by MT-hTer 47A, but not by general DNA damage (MMS). (E) Colocalization of telomeres with DNA damage response foci visualized by staining with an antibody against 53BP-1, a DNA damage protein (left three panels). In UMUC3 cells, introduction of a 47A MT-hTer construct induced a rapid DNA damage response and telomere uncapping response, including telomere dysfunction-induced foci, within 3 days. The 47A MT-hTer expressing cells showed more than 70% of DNA damage foci colocalization on telomeres, indicative of telomere-specific damage (telomere uncapping) (the bar plot in the right panel). Similar results were shown for colocalization of telomeres [file 1756-8935-1-4-S14.pdf]

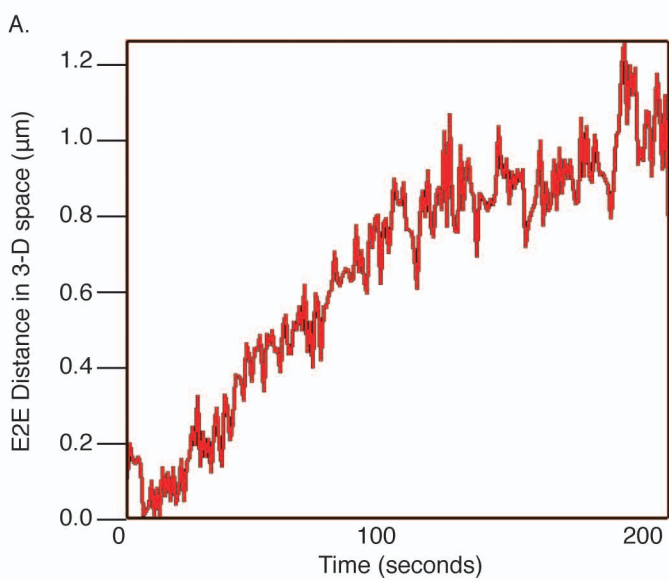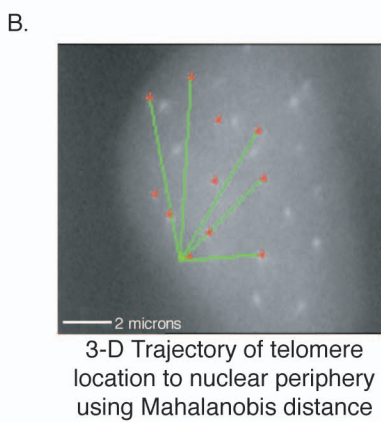

fig. S6

Supplement: Additional file 16 — Justified assessment of quantifying telomere motions by effective diffusion coefficients. (A) Averaged mean-square-distance (MSD) versus time for around 800 telomeres shows that telomere motion imaged within 200 seconds does not reach the plateau indicative of constrained motion. The plot shows the MSD curves for the telomeres and demonstrates that the MSD value does not decrease (saturate the volume of nuclear three-dimensional (3-D) space available for movement) over a 200-second time interval of observation. (B) A 3-D trajectory image at any z-section and any time of a UMUC3 nucleus shown to estimate the distance of each telomere to the nuclear periphery (indicated by the red lines). [file 1756-8935-1-4-S16.pdf]

H.

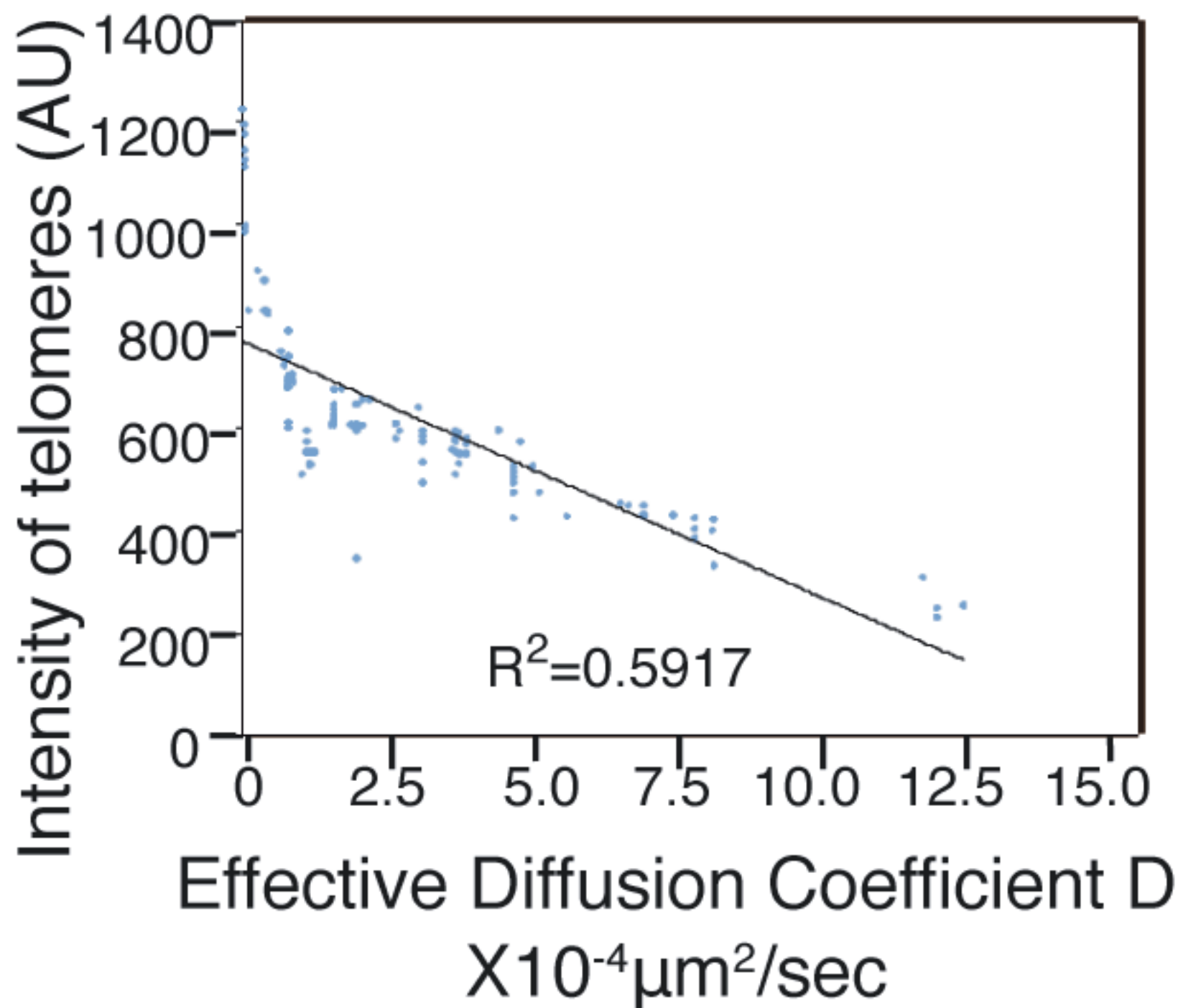

Supplement: Additional file 17 — Green fluorescent protein (GFP)-TRF1 labeled, parental UMUC3 clonal cells were imaged using OMX microscopy. Cells with similar intensity/AU under the same light exposure were chosen and their telomere motility (N = around 200) analyzed. The end-to-end (E2E) measurements of these telomeres were used to calculate their effective diffusion coefficients D (10-4 μm2/second) using Einstein's diffusion equation, E2E = √(6DT) (where T is the time in seconds). Each telomere's intensity was plotted against its D value to give a scatter plot indicative of the relationship between telomere length and motility. The linear regression line shows a value of R2 = 0.5917 in the graph. [file 1756-8935-1-4-S17.pdf]
