# Supplementary material for: Rapid telomere motions in live human cells analyzed by highly time-resolved microscopy
Source: Epigenetics Chromatin. 2008 Oct 27;1:4. doi: 10.1186/1756-8935-1-4 (PMC2585561; doi:10.1186/1756-8935-1-4)
Supplement: Additional file 9 — Quantifying telomere motions in live cells. Averaging of end-to-end (E2E) distances for quantitative measurement of telomere motion. The path of a moving particle (bottom left) is divided up into intervals of integer numbers of 1-second time points. For each 1-second increase in the interval considered, the number of intervals decreases by one; thus larger intervals have fewer samples and are more subject to stochastic variation. Lengths traveled during each interval are shown to the right of each figure. These lengths are averaged together (average length shown under the black line). When the average lengths from a particle undergoing unconstrained random diffusion are plotted, they scale with the square root of elapsed time. This procedure helps to reduce measurement noise, especially for short time differences. Telomeres tracked for N seconds are plotted for intervals up to N/2 seconds. [file 1756-8935-1-4-S9.pdf]

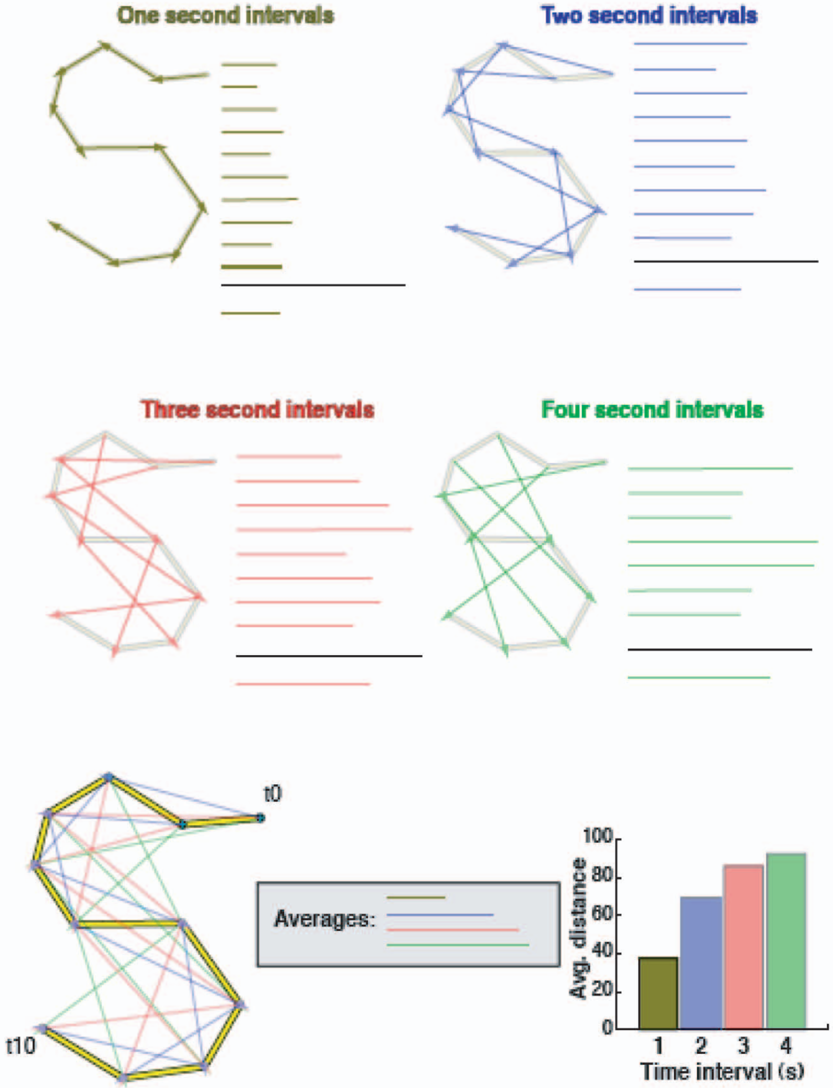
One second intervals
Two second intervals
Three second intervals
Four second intervals
t0
t10
Averages:
100
80
60
40
20
0
Avg. distance
1
2
3
4
Time interval (s)

fig. S3
