# Supplementary material for: Rapid telomere motions in live human cells analyzed by highly time-resolved microscopy
Source: Epigenetics Chromatin. 2008 Oct 27;1:4. doi: 10.1186/1756-8935-1-4 (PMC2585561; doi:10.1186/1756-8935-1-4)
Supplement: Additional file 15 — Sizes and diffusion coefficients of particles in the nucleus and of chromatin loci. (A) Copied from Gorisch SM, Lichter P, Rippe K. (2005): Dextran particles in different milieus. (B) Various other measurements of D in the nucleus. [file 1756-8935-1-4-S15.doc]

**Table S2**

Sizes and diffusion coefficients of particles in the nucleus and of chromatin loci

**A.** (Copied from Gorisch SM, Lichter P, Rippe K. (2005) [1]:

Dextran particles in different milieus.

__________________________________________________

Diffusion Coefficients Dextran MW Stokes Radius

(μm^2^ s^-1^) (kd) (nm)

__________________________________________________

2.86 in nucleoplasm 62 55.1

1.97  in cytoplasm 62 55.1

39 in water 62 55.1

__________________________________________________

_____________________________________________________________

In nuclei:

Particle/locus r_c_ (nm) D (μm^2^ s^-1^) Reference

_____________________________________________________________

Mx1-YFP 280 1.8·10^-4^ [2]

Cajal bodies 310 1.1·10^-4^ [2]

PML bodies 260 1.2·10^-4^ [2]

Nanospheres 150 4·10^-4^ [3]

Nucleoplasmic chromatin 240 1.3·10^-4^ [4]

Telomeras 230 1.8·10^-4^ [5]

Dense chromatin regions 180 4.8·10^-5^ [2]

1-Mb chromatin domain – 0.5–1.5·10^-5^ [6]

**B.** Various other measurements of D in the nucleus.

_______________________________________________________________________

Yeast telomeres D=4x10^-4^μm^2^ s^-1^ [7]

PNA probes of PML bodies D=1.8-5.8x10^-4^ μm^2^ s^-1^ [5]

Yeast lac sites D=5x10^-4^μm^2^ s^-1^ [8]

Subchromatin labels various cell lines D=0.1-1.25x10^-4^μm^2^ s^-1^ [4, 6]

Centromeres majority are stable [9]

Centromeres and telomeres **velocity** V=0.14μm/min [10]

Drosophila Lac labels D=0.1-1.3x10^-2^μm^2^ s^-1^ [11]

Yeast, SAGA labeled transcribed

genes D~2.5x10^-4^μm^2^s^-1^ [12]

**REFERENCES**

1. Gorisch SM, Lichter P, Rippe K: **Mobility of multi-subunit complexes in the nucleus: accessibility and dynamics of chromatin subcompartments.** *Histochem Cell Biol* 2005, **123:**217-228.

2. Gorisch SM, Wachsmuth M, Ittrich C, Bacher CP, Rippe K, Lichter P: **Nuclear body movement is determined by chromatin accessibility and dynamics.** *Proc Natl Acad Sci U S A* 2004, **101:**13221-13226.

3. Tseng Y, Lee JS, Kole TP, Jiang I, Wirtz D: **Micro-organization and visco-elasticity of the interphase nucleus revealed by particle nanotracking.** *J Cell Sci* 2004, **117:**2159-2167.

4. Chubb JR, Boyle S, Perry P, Bickmore WA: **Chromatin motion is constrained by association with nuclear compartments in human cells.** *Curr Biol* 2002, **12:**439-445.

5. Molenaar C, Wiesmeijer K, Verwoerd NP, Khazen S, Eils R, Tanke HJ, Dirks RW: **Visualizing telomere dynamics in living mammalian cells using PNA probes.** *Embo J* 2003, **22:**6631-6641.

6. Bornfleth H, Edelmann P, Zink D, Cremer T, Cremer C: **Quantitative motion analysis of subchromosomal foci in living cells using four-dimensional microscopy.** *Biophys J* 1999, **77:**2871-2886.

7. Hediger F, Neumann FR, Van Houwe G, Dubrana K, Gasser SM: **Live imaging of telomeres: yKu and Sir proteins define redundant telomere-anchoring pathways in yeast.** *Curr Biol* 2002, **12:**2076-2089.

8. Marshall WF, Straight A, Marko JF, Swedlow J, Dernburg A, Belmont A, Murray AW, Agard DA, Sedat JW: **Interphase chromosomes undergo constrained diffusional motion in living cells.** *Curr Biol* 1997, **7:**930-939.

9. Shelby RD, Hahn KM, Sullivan KF: **Dynamic elastic behavior of α-satellite DNA domains visualized in situ in living human cells.** *J Cell Biol* 1996, **135:**545-547.

10. Cheutin T, McNairn AJ, Jenuwein T, Gilbert DM, Singh PB, Misteli T: **Maintenance of stable heterochromatin domains by dynamic HP1 binding.** *Science* 2003, **299:**721-725.

11. Vazquez J, Belmont AS, Sedat JW: **Multiple regimes of constrained chromosome motion are regulated in the interphase Drosophila nucleus.** *Curr Biol* 2001, **11:**1227-1239.

12. Cabal GG, Genovesio A, Rodriguez-Navarro S, Zimmer C, Gadal O, Lesne A, Buc H, Feuerbach-Fournier F, Olivo-Marine JC, Hurt EC, Nehrbass U: **SAGA interacting factors confine sub-diffusion of transcribed genes to the nuclear envelope.** *Nature* 2006, **441:**770-773.
