# Supplementary material for: Rapid telomere motions in live human cells analyzed by highly time-resolved microscopy
Source: Epigenetics Chromatin. 2008 Oct 27;1:4. doi: 10.1186/1756-8935-1-4 (PMC2585561; doi:10.1186/1756-8935-1-4)
Supplement: Additional file 18 — Table 1 – A summary of the Komolgorov-Smirnov non-parametric comparison between histograms discussed in the text. A Komolgorov-Smirnov (K-S) score three times the error (standard deviation) is considered significant. Red scores indicate statistically significant different histograms, while the orange score is at the borderline for statistically significant difference between the two histograms under comparison. The first value in the right column is the K-S value between histograms, and the second value after ± is the bootstrap value (error) for each histograms. Hence, the table shows that there is significant difference when comparing histograms of parental or vector control cells with either heterochromatin, azide-treated cells, WT-hTER-treated cells, or 47A MT-hTer-treated cells (red-colored scores and orange score). Bootstrap analysis for all histograms within error bars of < 0.05, hence comparisons between histograms are valid. [file 1756-8935-1-4-S18.pdf]

Komolgorov-Smirnov nonparametric comparison between histograms

Parental telomeres and lentiviral-vector control telomeres	0.013 ± 0.031
Parental telomeres and azide-treated telomeres	0.076 ± 0.026
Parental telomeres and heterochromatin spots	0.517 ± 0.029
Lentiviral-vector control telomeres and WT-hTER treated telomeres	0.199 ± 0.027
Lentiviral-vector control telomeres and 47A MT-hTer treated telomeres	0.269 ± 0.025

K-S score between histograms

K-S error within each histogram

Supplementary table 1
